# Where the rubber meets the road: Emerging environmental impacts of tire wear particles and their chemical cocktails

**DOI:** 10.1016/j.scitotenv.2024.171153

**Published:** 2024-03-07

**Authors:** Paul M. Mayer, Kelly D. Moran, Ezra L. Miller, Susanne M. Brander, Stacey Harper, Manuel Garcia-Jaramillo, Victor Carrasco-Navarro, Kay T. Ho, Robert M. Burgess, Leah M. Thornton Hampton, Elise F. Granek, Margaret McCauley, Jenifer K. McIntyre, Edward P. Kolodziej, Ximin Hu, Antony J. Williams, Barbara A. Beckingham, Miranda E. Jackson, Rhea D. Sanders-Smith, Chloe L. Fender, George A. King, Michael Bollman, Sujay S. Kaushal, Brittany E. Cunningham, Sara J. Hutton, Jackelyn Lang, Heather V. Goss, Samreen Siddiqui, Rebecca Sutton, Diana Lin, Miguel Mendez

**Affiliations:** a US Environmental Protection Agency, Office of Research and Development, Center for Public Health and Environmental Assessment, Pacific Ecological Systems Division, Corvallis, OR 97333, United States of America; b San Francisco Estuary Institute, 4911 Central Ave, Richmond, CA 94804, United States of America; c Department of Fisheries, Wildlife, and Conservation Sciences, Coastal Oregon Marine Experiment Station, Oregon State University, Corvallis, OR 97331, United States of America; d Department of Environmental and Molecular Toxicology, School of Chemical, Biological and Environmental Engineering, Oregon State University, Corvallis, OR 97333, United States of America; e Department of Environmental and Molecular Toxicology, Oregon State University, Corvallis, OR 97331, United States of America; f Department of Environmental and Biological Sciences, University of Eastern Finland, Kuopio Campus, Yliopistonranta 1 E, 70211 Kuopio, Finland; g US Environmental Protection Agency, ORD/CEMM Atlantic Coastal Environmental Sciences Division, Narragansett, RI 02882, United States of America; h Southern California Coastal Water Research Project, 3535 Harbor Blvd, Suite 110, Costa Mesa, CA 92626, United States of America; i Environmental Science & Management, Portland State University, Portland, OR 97201, United States of America; j US Environmental Protection Agency, Region 10, Seattle, WA 98101, United States of America; k School of the Environment, Washington State University, Puyallup Research & Extension Center, Washington Stormwater Center, 2606 W Pioneer Ave, Puyallup, WA 98371, United States of America; l Interdisciplinary Arts and Sciences (UW Tacoma), Civil and Environmental Engineering (UW Seattle), Center for Urban Waters, University of Washington, Tacoma, WA 98402, United States of America; m Civil and Environmental Engineering (UW Seattle), University of Washington, Seattle, WA 98195, United States of America; n US Environmental Protection Agency, Center for Computational Toxicology and Exposure, Chemical Characterization and Exposure Division, Computational Chemistry & Cheminformatics Branch, 109 T.W. Alexander Drive, Research Triangle Park, NC 27711, United States of America; o Department of Geology & Environmental Geosciences, College of Charleston, Charleston, SC, 66 George Street Charleston, SC 29424, United States of America; p Washington State Department of Ecology, 300 Desmond Drive SE, Lacey, WA 98503, United States of America; q CSS, Inc., 200 SW 35th St, Corvallis, OR 97333, United States of America; r Department of Geology and Earth System Science Interdisciplinary Center, University of Maryland, College Park, MD 20740, United States of America; s Department of Environmental and Molecular Toxicology, Oregon State University, Corvallis, OR 97333, United States of America; t Department of Anatomy, Physiology, and Cell Biology, Department of Medicine and Epidemiology and the Karen C. Drayer Wildlife Health Center, University of California, Davis School of Veterinary Medicine, Davis, CA 95616, United States of America; u US Environmental Protection Agency, Office of Water, Office of Wastewater Management, Washington, DC 20004, United States of America; v GSI Environmental, Inc., Olympia, Washington 98502, USA

**Keywords:** Persistent pollutants, Emerging contaminants, Microplastics, Tire wear particles, 6PPD-quinone

## Abstract

About 3 billion new tires are produced each year and about 800 million tires become waste annually. Global dependence upon tires produced from natural rubber and petroleum-based compounds represents a persistent and complex environmental problem with only partial and often-times, ineffective solutions. Tire emissions may be in the form of whole tires, tire particles, and chemical compounds, each of which is transported through various atmospheric, terrestrial, and aquatic routes in the natural and built environments. Production and use of tires generates multiple heavy metals, plastics, PAH’s, and other compounds that can be toxic alone or as chemical cocktails. Used tires require storage space, are energy intensive to recycle, and generally have few post-wear uses that are not also potential sources of pollutants (*e.g*., crumb rubber, pavements, burning). Tire particles emitted during use are a major component of microplastics in urban runoff and a source of unique and highly potent toxic substances. Thus, tires represent a ubiquitous and complex pollutant that requires a comprehensive examination to develop effective management and remediation. We approach the issue of tire pollution holistically by examining the life cycle of tires across production, emissions, recycling, and disposal. In this paper, we synthesize recent research and data about the environmental and human health risks associated with the production, use, and disposal of tires and discuss gaps in our knowledge about fate and transport, as well as the toxicology of tire particles and chemical leachates. We examine potential management and remediation approaches for addressing exposure risks across the life cycle of tires. We consider tires as pollutants across three levels: tires in their whole state, as particulates, and as a mixture of chemical cocktails. Finally, we discuss information gaps in our understanding of tires as a pollutant and outline key questions to improve our knowledge and ability to manage and remediate tire pollution.

## Graphical Abstract

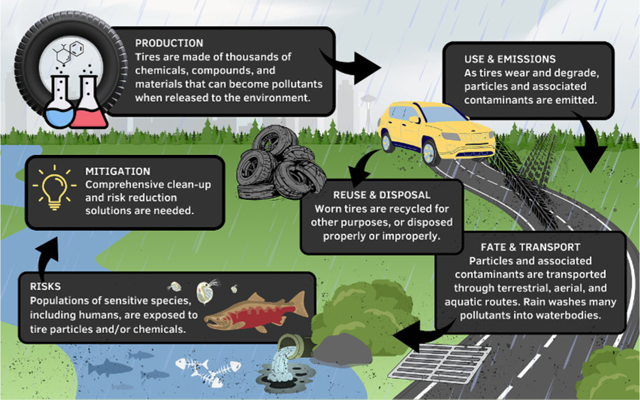

## 1. Introduction

Global dependence on tires produced from petroleum-based compounds, synthetic materials, heavy metals, and added chemicals, represents a persistent and complex environmental problem with only partial, and often-times ineffective, solutions. Used tires require storage space, are energy intensive to recycle, and end-of-life uses for tires (*e.g*., crumb rubber, pavements, combusted tires) generally continue to release pollutants as particles or leached chemicals, or both. Tires are a significant source of highly mobile, persistent microplastics (Moran et al., 2021; Brander et al., 2021) that are a major component of pollutants in urban stormwater runoff (Wik and Dave, 2009). Furthermore, recent research has demonstrated that tires are a source of previously unrecognized chemicals, some are highly toxic to aquatic organisms, and many of which are currently unknown or poorly described (Tian et al., 2021a; Siddiqui et al., 2022; Cunningham et al., 2022). Production and use of tires generate a suite of heavy metals and other contaminants that can be toxic alone or as chemical cocktails, which represent combinations of elements novel to the Anthropocene (*sensu* Kaushal et al., 2020). Given that roads are ubiquitous in developed nations (Ibisch et al., 2016), cover extensive areas in urban ecosystems (Elmore and Kaushal, 2008), and that road construction and traffic are increasing worldwide (Meijer et al., 2018), the impacts of tires are vast and are expected to increase globally. Roads can represent hot spots of tire pollutants and effective pathways of pollutants to aquatic, terrestrial, atmospheric, and groundwater resources (Cooper et al., 2014; Sommer et al., 2018; Uliasz-Misiak et al., 2022).

Here we synthesize recent research and data about the environmental and human health risks associated with tire production, use, and disposal. We discuss gaps in our knowledge about fate and transport, as well as the toxicology of tire particles and leachates. We examine potential management and remediation approaches for addressing exposure risks across the life cycle of tires and associated contaminants. We consider tires as pollutants across three levels: whole tires, tire wear particles, and as a mixture of chemical constituents. Finally, we outline key questions to expand our knowledge and ability to manage and remediate pollution from tires.

## 2. The composition of tires

Tires are constructed of multiple, highly engineered components, including tread, belts, inner liners, and sidewall, each designed to meet performance characteristics that together create durable, strong, reliable, and safe tires (USTMA, 2018) which are the same properties that ensure the persistence of tire particles and tire materials in the environment. Tires contain myriad materials and chemicals, many of which are proprietary (Tian et al., 2021b). Tires typically use metal mesh or textiles for structure and rubber for all other components. Tire rubber consists of complex proprietary formulations that vary among brands, tire types, and tire components (Hüffer et al., 2019; Kreider et al., 2010; Selbes et al., 2015; Wagner et al., 2018; Chibwe et al., 2022). In general, tire rubber consists of synthetic and/or natural rubber (40–60 %), fillers and reinforcing agents like carbon black and silica (20–25 %), process or extender oils (12–15 %), vulcanization agents like Zn and thiazoles (1–2 %), and other additives such as preservatives and processing aids (5–10 %) (Wagner et al., 2018). Tires contain approximately 50:50 ratio of natural to synthetic rubber; passenger car tires contain more synthetic rubber, while truck tires more natural rubber, and heavy-duty vehicles tires contain little or no synthetic rubber (Grammelis et al., 2021). Tires contain thousands of chemicals, including those deliberately added, such as N-(1,3-dimethylbutyl)-*N*′-phenyl-*p*-phenylenediamine (6PPD, DTXSID9025114; CAS 793–24–8), contaminants in manufacturing feedstocks, such as polycyclic aromatic hydrocarbons (PAHs), and weathering or transformation products as tires age (Tian et al., 2021b; European Tyre and Rubber Manufacturer’s Association, 2021; Kovochich et al., 2021). As a result, there is no standard chemical composition of tire wear particles. This creates challenges for monitoring and characterizing tire particles and tire-derived chemicals in environmental samples, for estimating tire microplastics fate, and for conducting ecotoxicology impact assessments. Determining the complete and quantitative chemical composition of tire rubber remains a critical research need.

## 3. Environmental and health impacts along the life cycle of tires

**Fig. 1.**
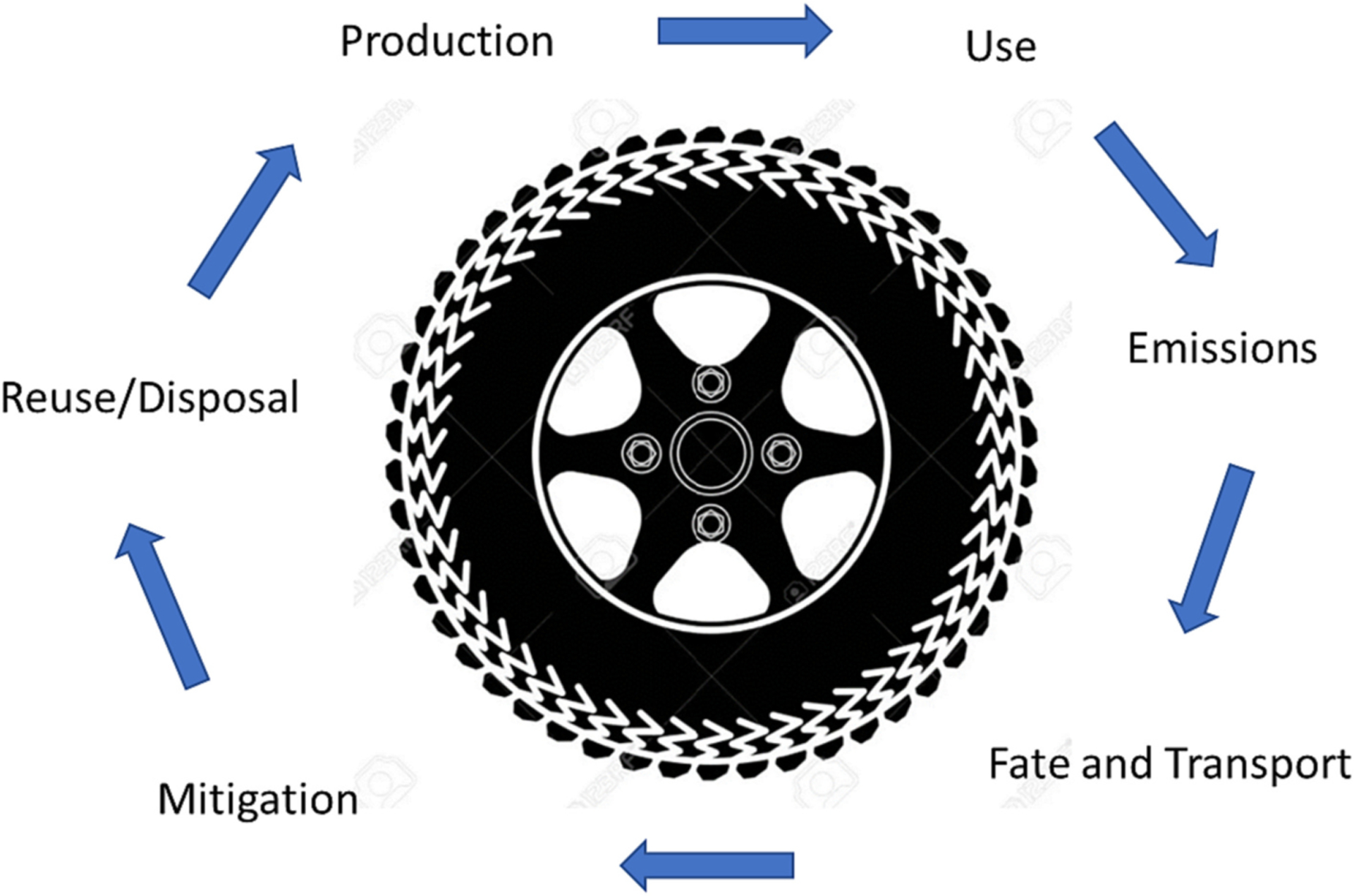
Life cycle of tires

The life cycle of a tire can be characterized by stages including a) raw materials and production of the whole tire, b) transportation of the tire to a destination, c) use on a vehicle, and d) end of life management through downcycling into non-tire products or disposal (Dong et al., 2021; Trudsø et al., 2022). Here, we examine these stages as a continuum along the life cycle of a tire (Fig. 1) where tires and their components are produced and used. In the process, energy and resources are consumed while particles and elements are emitted and transported through the environment across various pathways (Trudsø et al., 2022). At points along those pathways, there are potential mitigation approaches to reduce environmental impacts including reuse or disposal, and, in some cases, recycling into new tires and tire related products. Manufacturing tires requires copious water and electricity and produces nitrogen oxides (NOx), benzene, and PAHs (Dong et al., 2021). Each tire life cycle stage has multiple impacts on climate and acidification from energy use and production of CO_2_, ozone depletion, photochemical oxidation, and eutrophication from NOx production and use of ${\text{PO}}_{4}^{3-}$ in manufacturing (Dong et al., 2021; Sun et al., 2016).

### 3.1. Environmental and health impacts from the production of tires

Global demand for automobile tires is large and growing. In 2019 alone, 3 billion tires were produced globally (Ruwona et al., 2019; Dong et al., 2021), an amount that, if stacked on top of one another, would reach *ca*. 675,000 km, nearly twice the distance to the moon. Recent tire production in the EU is about 335 million annually (Torretta et al., 2015), while tire production was about 300 million in the US (USTM, 2022) and about 800 million in China (Dong et al., 2021). Tire production begins with acquisition of natural rubber for the tread, textiles and steel for the cord and belts, and chemicals such as carbon black, silicon dioxide, and clay (Dong et al., 2021). A significant environmental impact of tire production is from the cultivation of natural rubber which involves clearing native, diverse forests for growing monocultures of rubber trees. This type of agriculture is an especially important cause of deforestation in Asia and Africa (Pendrill et al., 2022). High resolution maps of southeast Asia show that rubber tree cultivation accounted for at least 4 million ha of deforestation since 1993, 2 million ha of which was lost since 2000, including 1 million ha of rubber plantations that have been established in high biodiversity areas (Wang et al., 2023).

Combining tire components during production emits carcinogens and radioactive compounds (*e.g*. radon-222 and carbon-14), contributes to stratospheric ozone depletion, and requires massive consumption of water and electricity, and land in the form of extraction of minerals and fossil fuels, and water (Piotrowska et al., 2019). Combined, the various chemical components and the particles create chemical cocktails (*sensu* Kaushal et al., 2018, Kaushal et al., 2020, Kaushal et al., 2022) of heavy metals (*e.g*., Zn), natural and synthetic rubber and plastics, hydrocarbons (*e.g*., PAHs), and traces of other chemicals (*e.g*., 6PPD) that can have negative effects on human health and the environment.

Manufacturing a single tire produces an estimated 243 g particulate matter to the air, 0.19 g ${\text{NH}}_{4}^{+}$ and 0.69 g suspended solids to the water (Sun et al., 2016). On average, 6 MJ of electrical energy, 45 L of water, and 0.02 kg of dissolvent are needed to manufacture one tire while yielding 0.5 kg of waste (Piotrowska et al., 2019). Extrapolating from an estimated 3 billion tires produced annually, tire manufacture may produce as much as 729 million kg particulate matter, 570,000 kg ${\text{NH}}_{4}^{+}$, and over 2 million kg suspended solids annually, while as much as 104 billion MJ energy may be consumed along with over 70 billion liters of water though, water and energy consumption could be reduced through existing technologies, including high-pressure steam to shorten vulcanization time and recovery of waste steam (Sun et al., 2016). Using the Intergovernmental Panel on Climate Change (IPCC) methodology, each tire requires over 333,000 kg CO2 Eq during production while 2116 kg of CO2 Eq are recovered by recycling a tire (Piotrowska et al., 2019). Some tire manufacturers are striving to reduce their factory carbon footprint and/or exploring new tire designs with longer lifespan or which could be retread like industrial tires, thereby saving significantly on the amounts of materials required for production (https://www.motortrend.com/features/future-tire-technology/?id=applenews).

### 3.2. Environmental and health impacts from the use of tires

Significant ecosystem impacts are from tire emissions of carbon dioxide and sulfur oxides or nitrogen oxides, which are greatest during the use stage of the life cycle and a function of fuel use of the vehicle (Piotrowska et al., 2019). Tire wear during use results in tire wear particle emissions into the environment. Vehicle tire wear produces an estimated 1.2–6.7 kg of particles, or about 10–16 % of the weight of the tire, over the lifetime of the tire (Sun et al., 2016; USTMA, 2021). Tire wear and evolution of tire wear products may be exacerbated by the heavier weight and increased acceleration and torque produced by EVs (Zhao et al., 2019). Tire microplastics from synthetic rubber tires are a major contributor of microplastic pollution to the environment (Kole et al., 2017; Sieber et al., 2020; Siegfried et al., 2017). Measurable and sometimes significant amounts of tire particles have been collected in air, aquatic environments, and organisms (Baensch-Baltruschat et al., 2020; Leads and Weinstein, 2019; Siegfried et al., 2017; Tian et al., 2017; Werbowski et al., 2021; Wik and Dave, 2009). For example, untreated stormwater runoff samples collected from San Francisco Bay watersheds contained up to 15.9 tire particles/L, almost 50 % of all microparticles in these samples (Sutton et al., 2019; Werbowski et al., 2021). Globally, tires may be one of the top sources of microplastics to the environment (Boucher and Friot, 2017; Kole et al., 2017; Sieber et al., 2020), with a pollutant mass exceeding the total environmental emissions of other pollutant classes like pharmaceuticals and pesticides (Wagner et al., 2018).

Tire emissions generally relate to vehicle weight, tire size, and distance traveled, with larger heavier vehicles (trucks) emitting more than small light ones. Higher traffic speeds result in increased generation of tire particles (Wang, 2017; Pohrt, 2019; Kwak et al., 2013; Foitzik et al., 2018). Particle generation from any specific vehicle or in specific roadway segments can vary depending on driving speed or style (*e.g*., urban stop/go *vs*. highway), road surface condition, type of contact (rolling *vs*. slipping) and temperature (Alexandrova et al., 2007; Knight et al., 2020; Kole et al., 2017). Based on relatively limited data, country-specific tire particle generation across size classes 10 nm to 1000 μm has been estimated to be as low as 0.23 kg/yr/capita in India to as high as 5.5 kg/yr/capita in the US due to its longer per-capita annual vehicle travel distances (Mennekes and Nowack, 2022; Baensch-Baltruschat et al., 2020; Councell et al., 2004; Wagner et al., 2018; Kole et al., 2017). Thus, approximately 1.7 million tons of tire wear particles are produced annually in the US based on 2021 population size. Where automobile and truck traffic are higher, production of particles may be significantly greater. Based on empirical and extrapolated data synthesized from Europe, Japan, China, Australia, Brazil, India, and USA annual global tire wear emissions, across size classes 10 nm −1000 μm, were estimated to be nearly 6 million tons (Baensch-Baltruschat et al., 2020).

Annually, about 800 million tires become waste material worldwide (Tsang, 2012). In the US, scrap tire generation in 2019 was about 260 million tires (USTMA, 2020). The global annual production of waste tires is estimated to reach 1.2 billion tons by the 2030s (Liu et al., 2020). Others estimate that, globally, 1.5 billion tires are discarded annually currently with an expected to increase to 5 billion tires by 2030 (Grammelis et al., 2021). Generally, waste tires remain in the region of their production. For example, only 3.1 % and 5.7 % of waste tires are exported from the US (USTMA, 2020) and the EU (Sienkiewicz et al., 2012), respectively. Waste tires are often recycled into various products, including outdoor products with high potential to disperse tire particles or tire-derived chemicals into the environment (Dabic-Miletic et al., 2021). For example, the majority of waste tire use in California, USA includes burning for fuel, crumb rubber production, and integration in civil engineering applications (Table 1). Worldwide, the fate of tires is similar with most going into energy production or recycled (Table 2). Tires are often downcycled into microplastic-containing products like tire crumb and tire buffings. Used tire processors separate tire rubber from tire structural components (*e.g*., steel belts) to produce various sized tire rubber pieces (Valente and Sibai, 2019) classified as buffings, ground, crumb, or aggregate, some of which contain or are entirely composed of microplastics. Products created from used tires include retreaded tires, tire-derived fuel, artificial turf infill, rubberized asphalt, shock absorption applications, landscaping mulch, playground and recreational areas, rubber-containing pavement seal coats, rubberized building and floor materials, railroad ties, and doormats (Dabic-Miletic et al., 2021). There are 12,000–13,000 synthetic turf fields in the US with 1200–1500 new installations annually (USEPA, 2019). Wear of turf fields, tracks, and other recreational areas where recycled tire crumb rubber is used can release tire microplastics into the environment (Wang et al., 2021).

## 4. Fate and transport

### 4.1. Cycling of tire particles in the environment

**Fig. 2.**
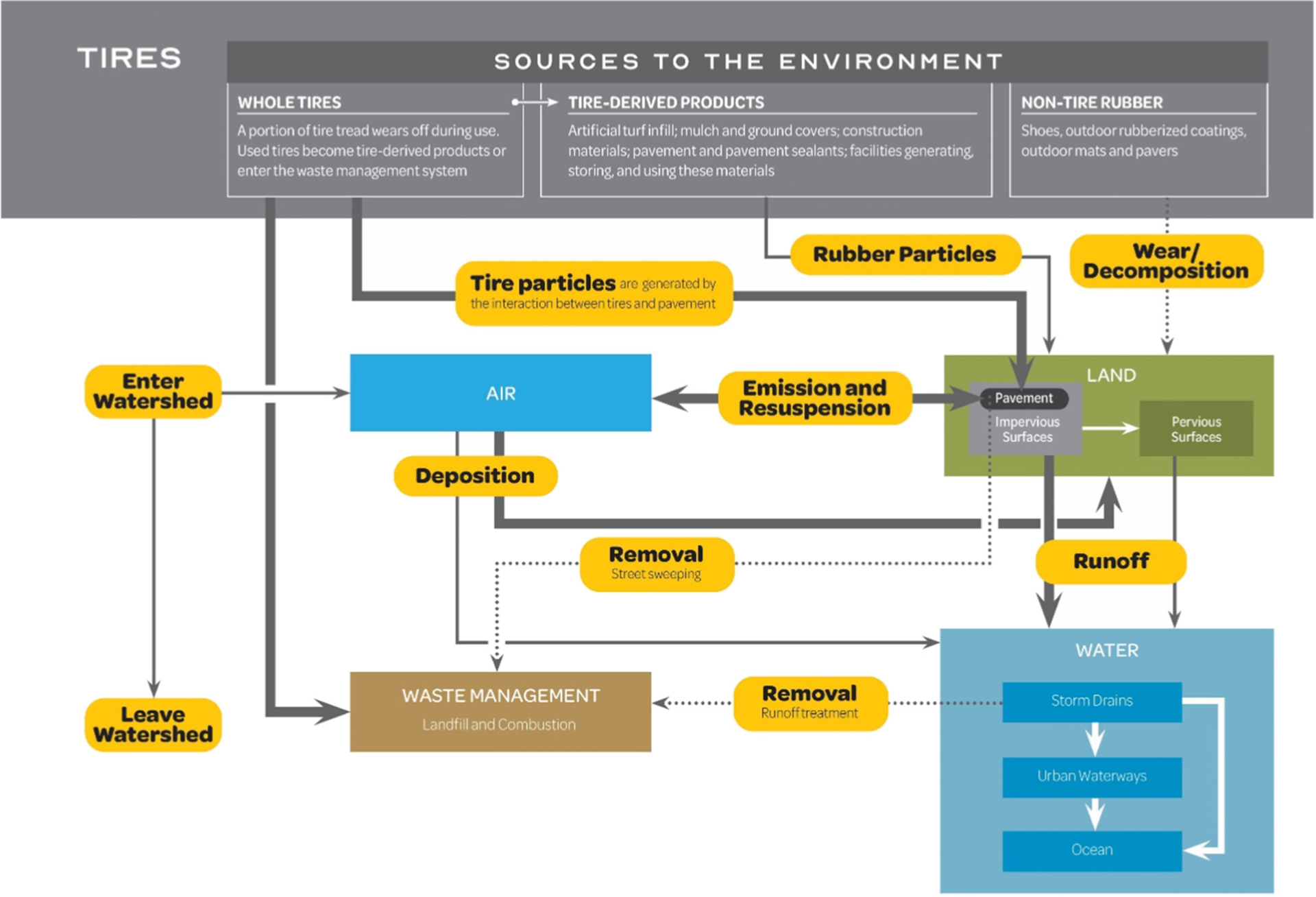
Conceptual model of the sources and pathways of rubber particles to urban stormwater. Major pathways are indicated by larger arrows, and dotted arrows represent minor pathways. The major source of rubber particles is tire wear, with tire-derived and non-tire rubber products representing smaller sources. Reprinted from Moran et al. (2021).

Studies of the fate and transport of tire microplastics and associated contaminants has been limited. A handful of studies have helped to characterize tire microplastics and affiliated leachate in the environment associated with urban runoff (Werbowski et al., 2021; Johannessen et al., 2021; Klöckner et al., 2020; Klöckner et al., 2021; Peter et al., 2018; Peter et al., 2020; Tian et al., 2021b). Data from Europe show that most of the mass of tire microplastics is deposited near roadsides, but that water and atmospheric pathways can move particles significantly farther (Baensch-Baltruschat et al., 2021; Evangeliou et al., 2020; Sieber et al., 2020; Sommer et al., 2018; Verschoor et al., 2016). Moran et al. (2021) conceptualized the sources and pathways of rubber particles to urban stormwater (Fig. 2).

Abrasion by pavement during vehicle use creates small tire wear particles. After their initial release to the air, tire wear particles may travel short (1–10 m) to long (km) distances prior to deposition, often promoted by localized effects of high-speed traffic. Roadway-derived particles (including tires) may compose >80 % of all microplastic air deposition, (Brahney et al., 2021). Notably, inhalation of atmospheric particulate matter is a critically important mechanism of human exposure to tire rubber microplastics and tire-derived chemicals. Recent studies documented substantial contributions of tire rubbers and associated chemicals to atmospheric particulate matter phases and associated human exposure risks *via* inhalation (Cao et al., 2022; Johannessen et al., 2022a; Zhang et al., 2021; Zhang et al., 2022).

Particles, especially those deposited on pavements, may be resus-pended, redistributed, or modified by vehicle traffic and environmental conditions. Vehicle tire abrasion grinds tire wear particles into pavement debris and soil, reducing particle size, encrusting particles with other road debris, and modifying particle shape (Kreider et al., 2010; Park et al., 2018) and chemical composition. Particles may also release tire related chemicals, including additive chemicals and their industrial and environmental transformation products, into air and water phases, or within biota that ingest particles (Peter et al., 2020; Tian et al., 2021b; Wagner et al., 2018).

For understanding particle transport, tire particles can be divided into three groups based on diameter: coarse (>2.5 μm), fine (<2.5 μm and > 0.1 μm), and ultrafine (<0.1 μm) (Wagner et al., 2018). Particle size governs transport distance, with fine and ultrafine particles most subject to long-distance aerial transport, depositing far from their sources (*e.g*., the Arctic, mountain wilderness) (Brahney et al., 2021; Evangeliou et al., 2020; Thornton Hampton et al., 2022; Wagner et al., 2018). Larger particles >10 μm typically deposit close to the point of emission (Cadle and Williams, 1978; Fauser et al., 2002; Kreider et al., 2010; Wagner et al., 2018). Available particle size distribution data indicate that most tire wear particle volume (and therefore mass) is in the coarse fraction (particles >50 μm), which deposit quickly from the source, landing on or close to pavements. While no studies show the full range of particle sizes, most tire wear particles are fine and ultrafine (Alves et al., 2020; Cadle and Williams, 1978; Fauser et al., 2002; Kreider et al., 2010). The smaller coarse and larger fine particles (between 1 μm and 10 μm), can be entrained into the atmosphere through mechanical processes, such as from the intense turbulence generated by high speed vehicle traffic (Brahney et al., 2021) and have atmospheric residence times of 8 days (<10 μm; “PM10”) to 28 days (<2.5 μm; “PM2.5”) (Evangeliou et al., 2020).

Fine (<10 μm) and ultrafine (<2.5 μm) tire particles comprise only a small fraction of the total mass of tire wear particles. However, they compose a large fraction of the total number of emitted particles, and their surface area might make them important vectors for tire chemical transport beyond the immediate roadside area. This is particularly true for smaller organisms and/or sensitive life stages. Due to limited data addressing tire particles across the full particle size distribution and the lack of surface area measurements for each size fraction, the role and importance of air deposition in tire particle and chemical transport within and between watersheds remains largely unknown (Moran et al., 2021).

Ultimately, tire wear particles are incorporated into soils and surfaces, washed off outdoor surfaces with runoff (Field et al., 2000; American Society of Civil Engineers, 1998), or washed out of the air by rainfall or snow. Particle wash-off from impervious surfaces (*e.g*., streets, sidewalks, roofs) is far more efficient than from permeable surfaces (*e.g*., lawns, gardens, agricultural fields) (Field et al., 2000; Pitt et al., 2008). Estimates of the portion of tire wear debris that is washed off of urban outdoor surfaces into urban runoff are highly variable and likely a function of many system and condition specific variables; *e.g*., 15–50 %, Wagner et al., 2018; 35 %, Blok, 2005; 80 %, Kennedy et al., 2002).

Modern urban and roadway drainage systems direct stormwater runoff directly (or indirectly *via* storm drains), untreated, into local water bodies. While tire particles may be temporarily retained in low points in stormwater collection systems under low flow conditions due to their density, turbulent flows during larger storm events will likely mobilize these particles and carry them into surface waters (Hoellein et al., 2019).

Tire particles may be washed by stormwater runoff into wastewater treatment systems. There are no current data on fate or volumes of tire particles specifically in wastewater treatment plants; however, data suggest that a high percentage of other microplastic particles transfer from the water into sewage sludge (Baensch-Baltruschat et al., 2021). Even with high removal rates, significant annual loadings of tire and road wear particles have been estimated in treated wastewater effluent in the UK (Parker-Jurd et al., 2021). Sewage sludge is typically incinerated, disposed of in landfills or spread on agricultural fields (Duis and Coors, 2016) where tire particles may remain in the soil or be mobilized and distributed by wind or by surface runoff to the aquatic environment (Duis and Coors, 2016; Baensch-Baltruschat et al., 2021).

Air transport and runoff may carry tire particles and associated chemicals into surface water drinking water sources (Johannessen and Metcalfe, 2022; Zhang et al., 2023). Drinking water treatment plants draw water from surface water, groundwater, and/or seawater, all of which may contain microplastics including tire particles (Collivignarelli et al., 2018). Drinking water treatment typically starts with screening and grit removal, followed by addition of alum to the raw water for coagulation, flocculation, and settling in tanks. Drinking water treatment plants are effective at removing small particles including 70–83 % of microplastics (<100 μm) with treatment by coagulation and membrane filtration showing high effectiveness (Nikiema and Asiedu, 2022; Pivokonsky et al., 2018). However, the level and types of drinking water treatment varies widely and effectiveness of drinking water treatment technologies to remove tire particles specifically is unknown.

Particle density affects particle transport. While tire rubber has a density of about 1.1–1.2 g/cm^3^ (Degaffe and Turner, 2011; Tang et al., 2006), slightly denser than water, tire wear particles collected near roads and generated by road simulators typically contain encrustations that increase density. Encrustations are diverse and consist of soil, pavement material, vehicle debris, road marking materials, *etc*. (Penkała et al., 2018; Thorpe and Harrison, 2008). Depending on the nature and quantity of encrustations, tire wear particle densities can range across 1.25–1.8 g/cm^3^ (Klöckner et al., 2021; Wagner et al., 2018). Higher density, encrusted particles will settle out more quickly, slowing transport and increasing potential for these particles to become sequestered on land or in aquatic sediment. Non-encrusted particles have sufficiently low density to transport readily in moving water (Tang et al., 2006). While some microplastics can be re-entrained into atmospheric pathways such as re-emission of previously deposited particles from the oceans to land (Allen et al., 2020), because tire wear particles are denser than seawater, the ocean to land pathway is unlikely for tire particles, except potentially the ultrafine particles.

### 4.2. Weathering of tires and chemical transformations

**Fig. 3.**
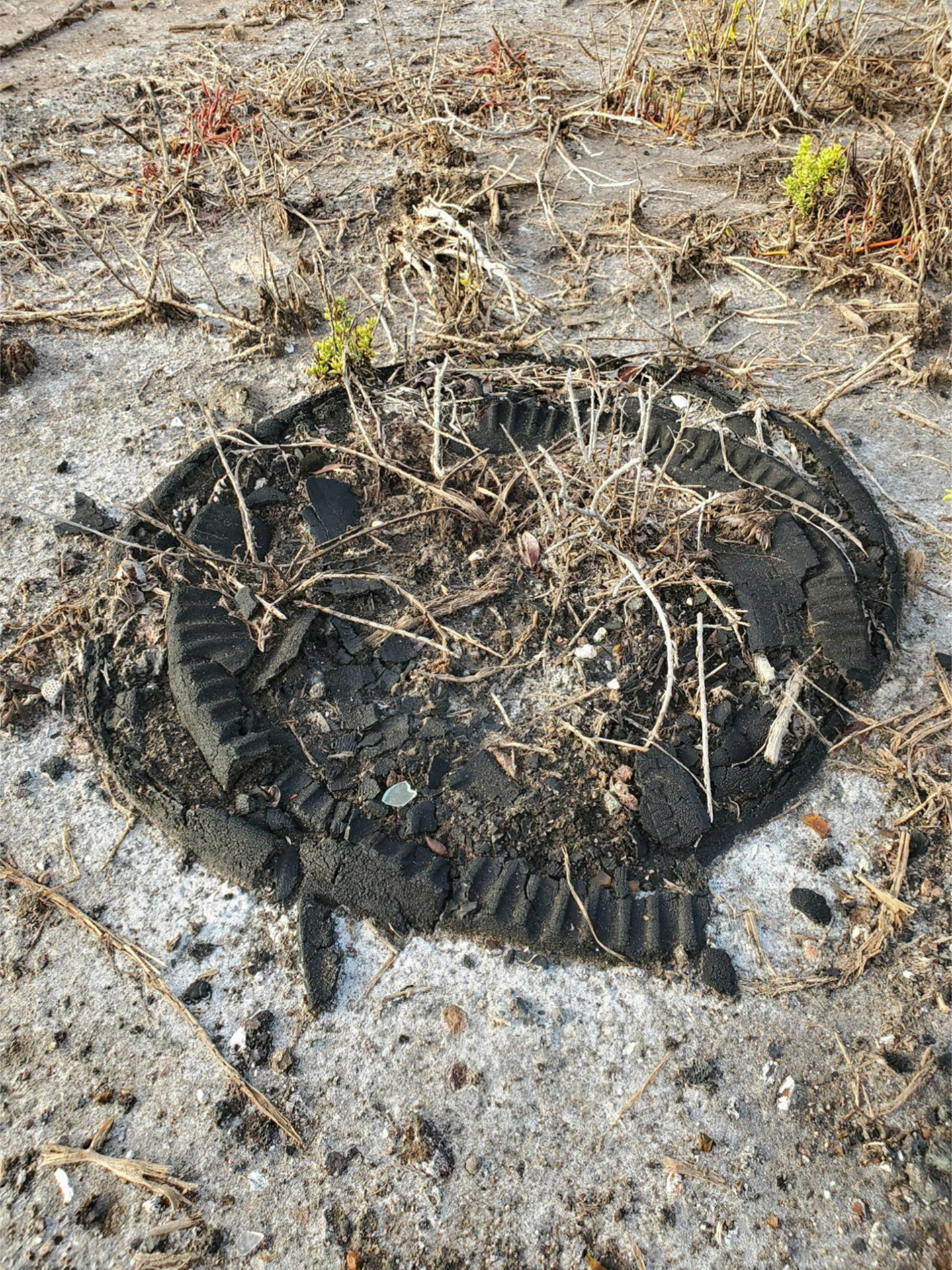
Degraded tire on an Oregon, USA beach (photo courtesy of James Kaldy)

Tire particles are relatively persistent in the environment but are subject to phototransformation, oxidation/reduction, hydrolysis, biotransformation, and mechanical degradation processes (Wagner et al., 2018; Fig. 3). Tire rubber is susceptible to ozonolysis, or the oxidative cleavage of carbon-carbon C=C double bonds, leading to cracks and crazing in the rubber (Newton, 1945). Tire particles are also subject to thermal degradation, sometimes reaching temperatures of up to 60 °C on hot asphalt (Higashiyama et al., 2016). Given tire rubber durability, physical modification other than by mechanical degradation should be relatively limited over short-term time scales (Wagner et al., 2018), although tire particles could potentially agglomerate with other particles in the environment (Moran et al., 2021). Currently, few studies have investigated the physical effects of weathering on tire wear particles, with most studies investigating bulk tire material or microplastics composed of other polymers.

Photoaging may have significant effects on the specific surface area of tire particles, thus facilitating chemical release. One study found a 55-fold increase in the surface area of tire particles obtained from a recycling plant, compared to a 13-fold increase in the surface area of polypropylene microplastics (Fan et al., 2021). The effects of environmental weathering processes on tire wear particle physical properties like specific surface area and particle deterioration remain an important knowledge gap. Additionally, there is little known about the role of microbiological processes in the degradation of tire particles and associated pollutants and the fate and transfer of pollutants carried into trophic chains.

Tire additives begin to transform during the production process and continue throughout the tire life cycle. Tire chemicals can transform and degrade at the surface and interior of the tire, the surface and interior of tire wear particles, and in the gaseous or dissolved phases following release from the tire polymer matrix. Oxidation reactions are common, including thermooxidation, photooxidation, ozonolysis, shear stress, biodegradation, and hydrolysis (Wagner et al., 2022). Chemical polarity can increase because of radical formation, hydrogen abstraction, oxygen addition, and formation of polar functional groups, including ketones and carbonyls (P. Fan and Lu, 2011; Rånby, 1989).

During rain, roadway runoff transports tire wear particles into receiving waters where ozonation and thermal degradation cease and photooxidation slows due to low temperature, low oxygen availability, and attenuation of ultraviolet light with depth and turbidity. When exposed to water, the dominant transformation processes for tire-related compounds include leaching, hydrolysis, and biodegradation. Depending on location, tire leachate compounds may be directly or indirectly (through storm drains) released into nearby waterways or transported to a treatment facility through combined sewer systems. Transformation products of tire additives have been detected in both the influent and effluent of wastewater treatment plants and municipal drinking water treatment plants (Johannessen and Metcalfe, 2022).

Transformation of tire compounds due to environmental weathering processes can drastically change the chemical signature and ecotoxicity of leachates. Transformation and degradation can lead to reduced or enhanced toxicity of certain compounds. Recent research efforts have put a spotlight on one product of 6PPD oxidation, 6PPD-quinone (N-(1,3-Dimethylbutyl)-*N*′-phenyl-*p*-phenylenediamine-quinone (DTXSID301034849; CAS 2754428–18–5) due to its lethal effects in coho salmon and other salmonids. This chemical is an ozonation product of the anti-ozonant 6PPD, which is intentionally added to tires to prevent ozonolysis and oxidation (Tian et al., 2021a; Brinkmann et al., 2022; Lo et al., 2023). Tires are typically composed of a mixture of several types of anti-aging additives including amine, phenolic, heterocyclic, and phosphite antioxidants (Castan et al., 2023).

### 4.3. Fate and transport of tire-related chemicals

Known chemical emissions from tires include benzothiazoles, PAHs, vulcanizers, antidegradants, aromatic and paraffinic oils, formaldehyde, and styrene-butadiene rubber polymers, along with heavy metals like Zn (Adachi and Tainosho, 2004; Cadle and Williams, 1978; Fauser et al., 1999; Klöckner et al., 2019; Kumata et al., 2011). A comprehensive list is shown in Supplementary Table 1. Despite their medium to long-term durability, both manufacture and aging of tire particles induce transformation of additive chemicals that generate complex mixtures of tire-derived environmental contaminants (Huang et al., 2021; Johannessen et al., 2022b; Peter et al., 2018, 2020; Tian et al., 2021a), which are subsequently transported by urban runoff into near-urban ecosystems (Johannessen et al., 2022b; Peter et al., 2018, 2020; Tian et al., 2021b; Halama et al., 2024). Particles transported to roadside soils, sediments, and surface water likely continue to release chemicals into aquatic environments and organisms that take up the particles. Supplementary Table 1 lists known or suspected chemical contaminants associated with tire rubbers.

Numerous chemicals are present at relatively high concentrations in aqueous leachates from tire materials, such as chemicals used or formed as byproducts during production, including metals (especially Zn, but also Al, Cu, Ni, Co, Mn); PAHs; vulcanization accelerators; crosslinking agents, corrosion inhibitors and their transformation products [*e.g*., benzothiazole (DTXSID7024586), diphenylguanidine (DTXSID3025178), hexamethoxymethyl melamine (DTXSID9027520)], anti-ozonates/oxidants (*e.g*., 6PPD); plasticizers (*e.g*., bisphenol A, phthalide, phthalates), organic compounds (*e.g*., dicyclohexylurea, 1-indanone)], and chemicals present at very low or non-detectable concentrations in tire material itself but that are apparently readily leachable and many unknowns or suspect chemicals (Capolupo et al., 2020; Chibwe et al., 2022; Halsband et al., 2020; Peter et al., 2020).

Urban environments harbor atmospheric cocktails of tire contaminants and other traffic-related pollutants, including nitrogen oxides and ozone from photochemical smog. In the atmosphere, volatile compounds and particles generated by volatilization and recondensation of semivolatile organic compounds are subject to reactions with atmospheric radicals (OH, O_3_, and NOx) in the presence of ultraviolet light. These reactions create the potential for chemicals from tire particles to generate carcinogenic nitrosamines formed by the reaction of parent molecules containing amine functional groups with nitrogen oxides (Johannessen et al., 2022a; Johannessen and Metcalfe, 2022). Other tire wear chemicals with implications for atmospheric transport include 2,2,4-trimethyl-1,2-dihydroquinoline (TMQ), 4,4′-dithiodimorpholine (DTDM), and tetramethyl thiram disulfide (TMTD), though their fate and transport are poorly understood (Johannessen et al., 2022a). The toxic tire wear compounds 6PPD-quinone and 4-aminodiphenylamine (4-ADPA) were detected in atmospheric samples collected at 5, 15, and 30 m from roads at levels estimated at 2.90 and 1.14 ng L – 1 for 6PPD-quinone and 4-ADPA, respectively, suggesting a potential human and wildlife health hazard (Olubusoye et al., 2023). While exposure is likely higher closer to roads, due to the long residence time of atmospheric tire particles (up to 28 days), tire chemicals can also travel long distances and contaminate distant environments (Evangeliou et al., 2020).

The magnitude of leaching from tire particles depends on numerous chemical and system-specific factors, including chemical properties (*e. g*., diffusivity, hydrophobicity, polarity, structure), tire material properties (*e.g*., chemical composition, aging/weathering or other surface alteration, particle size or surface area), and location and binding interactions of the chemical to the tire matrix. Additionally, leachate generation system variables also influence boundary conditions of mass transfer and thermodynamic equilibrium, such as liquid-to-solid ratio, time, mixing regime, temperature, and water phase composition, including pH, salinity, and presence of co-solvents, organic matter, or infinite sink conditions. Observed leachate concentrations of individual chemicals can vary widely across studies due to these many system variables. For example, changing temperature and turbulence/flow regimes were found to significantly affect tire leachate toxicity (Kolomijeca et al., 2020). More hydrophobic compounds, such as high molecular weight PAHs and 6PPD, were observed to preferentially leach and then sorb to sediments when leaching occurs in the presence of natural sediment (Unice et al., 2015). Leaching of Zn and other metals increases in acidic solutions and freshwaters (*versus* saltwater) due to the influence of pH and ionic strength on metal solubility (Capolupo et al., 2020; Selbes et al., 2015).

Chemical reaction and transformation in leachates also occur, and while transformation products are often observed in laboratory studies, it can be especially difficult to interpret temporal dynamics in complex mixtures and to represent environmental field conditions in the laboratory. Studies often report leachate concentrations and may also calculate an aqueous loading (μg chemical in solution/g tire) that accounts for the mass of tire in solution, or the liquid-to-solid ratio, used to generate the leachate (μg/L * g tire/L). However, this does not account for the mass of the chemical compound in the system, which is dependent upon both the tire mass and the rubber phase concentration of the chemical. For studies reporting the solid phase concentration (μg chemical in tire/g tire), the fraction (or %) of chemical leached into solution can be calculated (μg chemical in solution/g tire divided by μg chemical in tire/g tire * 100 %). To illustrate, data from four studies on benzothiazole leaching, a compound frequently reported in tire leachates, is compiled in Table 3. Tire material loading in the leaching studies (Table 3) is at least 10× but usually several orders of magnitude higher than water concentrations of tire particles observed in the environment (*i.e*., <0.1 g/L; Wagner et al., 2018). Halsband et al. (2020) found similar leachate concentration of benzothiazole after 48 h and 14 d at a concentration of 100 g tire/L (liquid:solid ratio 10 L/kg), indicating rapid release and approach to batch equilibrium. Aging and weathering of material in the environment can reduce the pool of leachable chemicals like benzothiazole (Unice et al., 2015), making certain organic compounds like (N-cyclohexylbenzothiazole-2-sulfenamide (CBS), N-(1,3-dimethylbutyl)-*N*′-phenyl-1,4-phenylenediamine (6-PPD), and 1,3-diphenylguanidine (DPG) less practical as tire pollutant markers in environmental samples.

### 4.4. Monitoring fate and transport of tire wear particles in the environment

Stormwater is considered a significant pathway for tire wear particles to enter aquatic systems (Wei et al., 2023). However, few studies have rigorously monitored tire wear particles in urban runoff. Most tire wear particles are smaller than <220 μm (Cadle and Williams, 1978; Fauser et al., 2002; Kreider et al., 2010). For example, in contrast to stormwater microplastic monitoring studies that employed a 300 μm filter (Sutton et al., 2019; Werbowski et al., 2021), monitoring in the San Francisco Bay area (California, USA) revealed that black, rubbery tire particles in untreated urban stormwater could pass through a smaller 125 μm pore filter (Moran et al., 2021). Even where environmental sampling methods do not exclude particles <220 μm (*e.g*., sediment samples), common density-based methods for separating microplastics from environmental samples often exclude denser tire wear particles (Klöckner et al., 2019).

Some tire chemical ingredients such as benzothiazoles, styrene-butadiene rubber, and Zn have been used as markers for the environmental presence of tires and occasionally as the basis for quantification of tire particles in environmental samples (Adachi and Tainosho, 2004; Cadle and Williams, 1978; Fauser et al., 1999; Klöckner et al., 2019; Kumata et al., 2011). While the markers have provided valuable insights on tire wear particle transport, due to other environmental sources, the high variability among tire formulations, and the potential leaching of marker chemicals from the tire matrix, recent analyses suggest such methods are unreliable for quantification of tire wear particles in environmental samples (Rauert et al., 2021; Wagner et al., 2018).

Both on-road and laboratory road-simulator-generated tire particles have similar, bimodal particle size distributions with peaks in both the ultrafine and coarse particle size groups (Cadle and Williams, 1978; Dahl et al., 2006; Fauser et al., 2002; Gustafsson et al., 2008; Kreider et al., 2010), making it difficult to measure particles across the size distribution by a single method (Wagner et al., 2018). For example, air quality monitoring typically focuses on particle sizes <10 μm (PM10) or < 2.5 μm (PM2.5) (*e.g*., Cadle and Williams, 1978; Dahl et al., 2006; Fauser et al., 2002), while aquatic and terrestrial monitoring typically focuses on particles >10 μm (*e.g*., Kreider et al., 2010).

Because tire wear particles are denser than fresh or estuarine water, there is high likelihood for them to become entrenched in aquatic sediments, making accurate sampling of tire particles in water difficult, though non-encrusted tire particles may be readily mobilized in flowing water (Tang et al., 2006). Thus, sediment sampling may be necessary to fully assess tire particle pollutant loads in streams or rivers.

### 4.5. Ecotoxicological effects

Tire particles can be ingested or respired, and toxicity is likely size-dependent and/or influenced by chemical composition (*e.g*., additives, associated pollutants, influence of weathering) and abiotic factors (Wagner et al., 2018). Tire particles likely behave similarly to other types of micro- and nanoplastics (MNPs), and a handful of studies, mainly in model aquatic organisms, indicate that mechanisms elicited by tire wear particle exposure are similar to other MNPs (Cunningham et al., 2022; Siddiqui et al., 2022). However, much remains unknown about absorption, distribution, metabolism, and excretion (ADME) processes across taxa, including humans. Additionally, no published studies have examined the difference in toxicity between new and weathered particles, tire particle mixtures from different tire types and brands, or the combination of tire, brake, and road wear that most realistically represents actual emissions to the environment.

Once entrained in sediments and soils, tire wear particles may serve as a source of adverse effects to benthic and soil populations, communities, and ecosystems. Consequently, tire particles and associated chemical additives have biological and larger scale ecological effects across the range of species and populations, types of communities and ecosystems, characteristics of the chemical additives, extent of particle weathering, and route of chemical exposure. For example, differing tire particle effects, such as 6PPD-quinone toxicity to some salmonids (McIntyre et al., 2021; Tian et al., 2022), have been found among matrices, such as sediment, water, and food (Khan et al., 2019; Redondo-Hasselerharm et al., 2018) and from both particles and additive leachates.

In general, MNPs contribute to food dilution, a phenomenon of reduced growth following ingestion due to either a false sense of satiation on the part of the organism, stress interfering with swimming and prey capture ability, or reduction of nutrient absorption (Siddiqui et al., 2022, Cunningham et al., 2022, Panko et al., 2013, Brander et al., 2024, Thornton Hampton et al., 2022), as well as oxidative damage if small enough (< 10 μm estimated) to translocate (Mehinto et al., 2022). Exposure to tire particles has been shown to result in differences in behavior, development, and other sublethal effects (Cunningham et al., 2022; Magni et al., 2022; Siddiqui et al., 2022; Sheng et al., 2021). Recent studies also indicate that nanoplastics (defined as particles 1 to 1000 nm; Gigault et al., 2018; Cunningham et al., 2023) may be able to translocate from the gut to other tissues and in some cases may be able to translocate into cells (Brander et al., 2024, Coffin et al., 2022, McIlwraith et al., 2021). Markers of oxidative stress have been demonstrated to increase following exposure to tire particles (Sheng et al., 2021; Shin et al., 2022). However, an increase in antioxidant defense may indicate an adaptive response to the presence of the tire particles. In episodic exposure to tire particles, mummichog (*Fundulus heteroclitus*) showed an adaptive response wherein DNA damage and repair were both increased, and antioxidant response mechanisms were altered in a dose-dependent manner (LaPlaca et al., 2022). To date, most studies on tire particles have focused on leachates, rather than particles, or have examined the combined toxicity of particles and heavy metals such as Zn (*e.g*., Yang et al., 2022). Differentiating the effects of particles themselves from the effects of harmful chemical leachates such as heavy metals is complex, and more research is needed to determine which variables cause oxidative stress and whether this is an adaptive *versus* a toxicological response.

Aqueous leachates of laboratory-generated tire tread particles and tire crumb are complex mixtures of chemicals, many of which have toxic effects (Capolupo et al., 2020; Celeiro et al., 2021; Unice et al., 2015). Several cationic metals associated with tire particles, including Zn, Fe, Cr, Pb, Ni, Cu, Ti, Sr, Ba, Se, Cd, and Pb, are routinely used materials in tires, brakes, catalytic converters, and road asphalt and are commonly detected in tire and road dust (Adamiec et al., 2016; Hong et al., 2020), potentially causing toxic effects in aquatic ecosystems. While there is a lack of direct information concerning TWP toxicity, there is toxicity information on the constituents of TWP such as heavy metals and PAHs. This information can be extrapolated, but matrix effects, biological uptake, and particular speciation of adjuvants/leachates may influence the toxicity of the individual toxicants in the TWP matrix. Zn has been identified as a major toxicant in tire particles and associated leachates (Liu et al., 2022; Wik et al., 2009; Yang et al., 2022) because ZnO and ZnS are added to tires during the vulcanization process (Adamiec et al., 2016). Zn levels as low as 0.65 μg/L and Cd levels as low as 0.56 μg/L can cause significant declines in marine copepod egg production, which is linked to population-level effects (Fisher and Hook, 2002). In addition, Ni can affect population structure in the estuarine copepod, *Eurytemora affinis* (30 μg/L for males and 50 μg/L for females) (Zidour et al., 2019). Other cationic metals such as Al and Cu, also found in tire particles, have exhibited population-level effects in a variety of aquatic organisms (Cleveland et al., 1991; Cribiu et al., 2020; Zidour et al., 2019). Because tires are not the only source, the proportional contribution of cationic metals from tires to the environment, remains a critical data gap.

Soils and water bodies near major roadways are typically contaminated with elevated levels of heavy metals, and concentrations are often positively correlated with increasing traffic and/or industrial activity (Aslam et al., 2013; Alsbou and Al-Khashman, 2017; Ghosh and Maiti, 2018). Chronic heavy metal exposure is known to cause ecotoxicological effects in wildlife including developmental abnormalities, neurotoxicity, and reduced reproduction and survival (Redondo-Hasselerharm et al., 2018; Capolupo et al., 2020; Ding et al., 2020; Sheng et al., 2021; Simon et al., 2021; Leifheit et al., 2022; Yang et al., 2022). For most studies, toxicological effects cannot be definitively linked to heavy metals alone due to the variability and complexity of the detected chemical constituents (Capolupo et al., 2020; Ding et al., 2020). However, a recent study by Yang et al. (2022) employed a Toxicity Identification Evaluation (TIE) approach to identify Zn as the causative agent for mortality in the marine copepod, *Tigriopus japonicus*, following exposure to tire particle leachate. Different experimental conditions (*e.g*., temperature, pH, soil organic content), differences in manufacturing or weathering among tire materials, and/or species sensitivity may influence bioavailability of heavy metals and alter toxicity profiles.

In addition to heavy metals, the toxicity of tires has often been attributed to non-polar organic compounds, such as PAHs (Wik and Dave, 2006), created by the incomplete combustion of organic matter and which are found in tires, crude oil, and gasoline (Hussain et al., 2018). The amounts and types of PAHs vary among tires (Sadiktsis et al., 2012). PAHs are leached from tire particles and other tire debris in water, soil, and biological fluids (Marwood et al., 2011; Panko et al., 2013; LaPlaca and van den Hurk, 2020) and can persist in the environment (Shuttleworth and Cerniglia, 1995) or transform into other potentially toxic compounds (Lundstedt et al., 2007). Much of the toxicity observed in leachate and tire debris exposures has been attributed to PAHs (Wik and Dave, 2006; McIntyre et al., 2014; Wu et al., 2014); however, the proportion of PAHs in the environment that originates from tires is unknown. Observed abnormalities from PAH exposure are like those seen in fish exposed to tire debris (McIntyre et al., 2014). PAHs and their metabolites can cause carcinogenic and non-carcinogenic impacts (Martins et al., 2013; Martins et al., 2015). For example, uptake of PAHs released from tire particles (Stephensen et al., 2003; LaPlaca and van den Hurk, 2020) can cause developmental defects and mortality in fish (Incardona et al., 2004; Fallahtafti et al., 2012; Incardona et al., 2012; Philibert et al., 2016) and PAH exposure can cause immunotoxicity, cardiotoxicity, and impact reproduction in wildlife (Honda and Suzuki, 2020).

The toxicity of PAHs is photo-enhanced by sunlight. For example, Pelletier et al. (2000) found potential population level effects from PAHs and UV light exposures in bivalves that had bioaccumulated 5000 μg PAH/g lipid (Pelletier et al., 2000). Bellas and Thor (2007) reported adverse effect levels for the marine calanoid copepod *Acartia tonsa* for pyrene and UV exposures, with concentrations that affect 50 % of the organisms (EC50s) of 16 μg/L (egg production rate) and 8.3 μg/L (recruitment rate). Environmental mixtures of PAHs can affect reproductive endpoints in mussels at tissue concentrations as low as 60 μg/Kg dw (Ruiz et al., 2011). PAHs decreased nauplii and copepodite production in an estuarine copepod (*Schizopera knabeni*) at sediment concentrations as low as 38 μg/Kg dw. Because of the many sources of PAHs in the environment, distinguishing the contribution from tires remains an important data gap.

In addition to PAHs, many other organic compounds exist in tires (Supplementary Table 1). Alkylphenols, including bisphenol A (BPA: DTXSID7020182), and other related alkylphenols such as octyl- and nonyl-phenol and alkyl-phenolic ethoxylates, have also been found in tire particles and associated leachates. These compounds are known endocrine disruptors in vertebrates, including several fish species (*e.g*., medaka, fathead minnow, sheepshead minnow, and rainbow trout) at concentrations as low as 100 μg/L (David et al., 2019) and are suspected to cause toxic effects on larvae, molting, and metamorphosis in invertebrates such as American lobster (*Homarus americanus*) (Laufer et al., 2013). 6PPD-quinone (DTXSID301034849), the degradate of 6PPD, an antiozonant used in tires, is highly toxic to juvenile Coho salmon (*Oncorhynchus kisutch*) (24 h LC50 of 0.095 μg/L) an ecologically, economically, and culturally important fish (Tian et al., 2022). Chinook salmon (*Oncorhynchus tshawytscha*) also suffer mortality from 6PPD-quinone, though juvenile coho were 3 orders of magnitude more sensitive than juvenile Chinook; 24-h median LC50 estimates were 41.0 *versus* 67,307 ng/L, respectively (Lo et al., 2023). Notably, coho LC50 was 2.3-fold lower than previously reported for adult coho (95 ng/L), emphasizing age-related differences in sensitivity (Lo et al., 2023). Brook trout (*Salvelinus fontinalis*) and rainbow trout (*Oncorhynchus mykiss*) also showed high sensitivity to this compound with reported 24 h LC50s of 0.59 and 1.96 μg/L, respectively (Brinkmann et al., 2022). The mode of action for 6PPD-quinone toxicity to salmonids remains unknown, as other species such as arctic char (*Salvelinus alpinus*), white sturgeon (*Acipenser transmontanus*), zebrafish (*Danio rerio*), and invertebrates (*Daphnia magna* and *Hyalella azteca*) are far less sensitive (Hiki et al., 2021; Brinkmann et al., 2022; Varshney et al., 2022). Studies on the aquatic toxicity of 6PPD-quinone are summarized in Table 4 and 6PPD-quinone occurrence in the environment is shown in Table 5.

Aside from their chemical composition, differences in tire particle size and shape (*e.g*., aspect ratio) may have varying levels of biological effects, as is the case for other types of MNPs (*e.g*., Schell et al., 2022). Overall, smaller MNPs can induce more significant biological effects compared to larger ones (Mehinto et al., 2022; Thornton Hampton et al., 2022). This may be because of their ability to disrupt epithelial barriers and affect tissue viability, recently demonstrated by Donkers et al. (2022) using a variety of different material types and sizes (nylon microfibers, tire particles, polystyrene, polyethylene). This phenomenon is size rather than material dependent, with particles <10 μm having the greatest propensity to translocate. Across plastic pollution in general, particles may be ingested but don’t appear to bioaccumulate (Gouin, 2020). Tire particles and their leachates are subject to influence from variation in conditions such as salinity, temperature, and pH that can lead to changes in particle chemistry and behavior, as well as toxicity. Abiotic variability is also known to influence particle behavior such as agglomeration, which plays a large role in particle fate and transport (Chang et al., 2022; Shupe et al., 2021). At increasing salinities for example, inland silverside (*Menidia beryllina*) and mysid shrimp (*Americamysis bahia*) both exhibited greater alterations in swimming behavior following exposure to nano-size tire particles (< 1 μm) and leachate (Siddiqui et al., 2022). Comparatively, the same organisms exhibited greater behavioral changes in the lowest salinity following exposure to micro-size tire particles (1–20 μm) (Siddiqui et al., 2022). In a study on fathead minnows *(Pimephales promelas*), temperature and mechanical stress had a significant negative effect on tire leachate toxicity; however, UV-A treatment of the tire particles (~ 1 mm) and range of pH conditions studied (8.2–8.6) did not alter toxicity (Kolomijeca et al., 2020). Future studies on varying abiotic factors on tire particle toxicity should consider incorporation of abiotic factors into their design since research on how these factors affect tire particle toxicity is largely unexplored to date.

Organisms can vary significantly in their response to tire particles (Table 6). For example, in estuarine organisms exposed to tire particles during the first few days of life, reduced growth was observed in larval fish (*Menidia beryllina*) and juvenile mysid shrimp (*Americamysis bahia*) (Siddiqui et al., 2022). Similarly, in the freshwater species *Danio rerio*, growth was reduced following exposure to tire particles (Magni et al., 2022). However, this trend does not hold across all species; *Daphnia magna* growth was not impacted in the same study, but fecundity was reduced due to internalization of tire particles (Magni et al., 2022). Another study with *D. magna* showed particle-based enhancement of toxicity, with greater toxicity from nanosized (<1 μm) in comparison to microsized (1–20 μm) tire particles; specifically, at high concentrations of nano and micro-sized tire particles, developmental deformities were observed in *D. rerio* as was mortality in both *D. rerio* and *D. magna* (Cunningham et al., 2022).

In experiments that evaluated the reproduction of *D. magna*, 15 and 150 mg/L of tire rubber particles significantly reduced the numbers of daphnids and their neonates (Schell et al., 2022). Furthermore, Magni et al. (2022) reported a Lowest Observed Effect Concentration (LOEC) of 9.8 mg/L for the same species exposed to TWPs. However, in 48 h tests with two levels of feeding and three types of tire materials, at concentrations up to 10,000 particles/L, reproduction of marine copepods *Acartia tonsa* and *Temora longicornis* was unaffected (Koski et al., 2021). Reproduction of the daphnid *Ceriodaphnia dubia* over 9 days was the most sensitive to tire rubber leachate exposure, with an EC50 of 10 mg/L (Wik et al., 2009). When the rotifer (*Brachionus plicatilis*) was exposed to tire particle leachate, a No Effect Concentration (NOEC) of 200 mg/L (0.2 g/L) was found (Shin et al., 2022). In experiments conducted with the mussel *Mytilus galloprovincialis,* gamete fertilization was affected by tire rubber leachates (Capolupo et al., 2020), a result attributed to a reduced motility of spermatozoa, possibly caused by leached metals (Capolupo et al., 2020). For the freshwater amphipod *Hyalella azteca*, Khan et al. (2019) found that neonate production, mortality, and growth, were all significantly impacted by TWP (500 to 2000 particles/mL) after a 21-d exposure. Additional studies with the amphipod *H. azteca* (Halle et al., 2021) with a concentration up to 250,000 particles/L, showed no significant effects on the reproductive output. In experiments using tire and road wear particles spiked in sediment (10,000 mg/Kg) or its elutriate, no reproductive endpoints were affected in the amphipod *H. azteca* or *C. dubia,* (Panko et al., 2013), and only slight growth effects were demonstrated in the insect midge *Chironomus dilutus* larvae and freshwater fish *Pimephales promelas*. However, the authors report reproductive measures in the *C. dubia* and *H. azteca* controls were also significantly reduced, most likely due to turbidity. This study is one of the few available investigations where abrasion of tires was mimicked to produce the TWP (Panko et al., 2013). The lack of effects in sediment exposures was corroborated in studies using several sediment organisms (Redondo-Hasselerharm et al., 2018; Carrasco-Navarro et al., 2022; Schell et al., 2022).

Reported adverse effects of TWPs on soil invertebrates are highly variable for oligochaetes, annelids, and nematodes (Ding et al., 2020; Pochron et al., 2017, 2018; Zhao et al., 2011). For example, Pochron et al. (2017) found that earthworms (*Eisenia fetida*) exposed to crumb rubber contamination in soil experienced reduced weight gain by 14 %; however, earthworm survivorship or stress response to heat and light was not impacted. Furthermore, crumb rubber contamination did not inhibit microbial respiration rates despite soils containing toxic concentrations of Zn (Pochron et al., 2017). In a similar study, exposure to virgin crumb rubber reduced weight gain by 14 % while aged crumb rubber did not have the same effect. However, exposure to aged crumb rubber reduced earthworm survival time during a stress test by 16 % relative to the survival time for worms in clean soil (Pochron et al., 2018). Ding et al. (2020) found that TWPs decreased survival of the soil worm (*Enchytraeus crypticus*) by >25 % and reproduction by >50 %. Tire particle exposure disturbed the microbiota of the worm’s guts causing an enrichment of microbes associated with opportunistic pathogenesis, suggesting that TWPs might impact soil biota and microbiota. Ding et al. (2023) found that *E. crypticus* avoided consuming TWP but that tire leachates exerted toxic effects on gut microbiota, intestinal histopathology, and metabolites at environmentally relevant concentrations and that toxicity varied with brand of tire. Zhao et al., 2011 studied the effects of waste crumb rubber in soils on nematode abundance, community structure and soil characteristics and found significant decreases in the numbers of plant parasitic nematodes, the most abundant trophic group. Furthermore, crumb rubber exposure decreased Shannon’s diversity index, evenness, and plant parasite index of soil nematodes, but increased dominance and maturity indexes. In a study exposing enchytraeid worms (*Enchytraeus crypticus*), springtails (*Folsomia candida*), and woodlice (*Porcellio scaber*) to TWPs (<180 μm) in soil and food at concentrations of 0.02 %, 0.06 %, 0.17 %, 0.5 %, and 1.5 % (*w*/w), Zn was again found to be the dominant metal and organic contaminants included benzothiazole, pyrene, chlorpyrifos, HCB, methoxychlor, and BDE (Selonen et al., 2021). At the highest test concentration in soil (1.5 %), TWPs decreased *F. candida* reproduction by 38 % and survival by 24 %, and acetylcholinesterase (AChE) activity of *P. scaber* by 65 %. In food, the 1.5 % test concentration of TWPs reduced *F. candida* survival by 38 % (Selonen et al., 2021). Altogether, the highly variable outcomes of the studies may be due to differences in particle size and chemical composition, and/or differences in species sensitivity, but suggest that TWPs can adversely affect soil invertebrates at concentrations found at roadsides in the environment (Selonen et al., 2021).

### 4.6. Population, community, and ecosystem effects

Population, community, and ecosystem level effects are important measures of the impacts of tires as pollutant as they integrate individual toxicological effects and are often measures of wide-scale change. Species especially sensitive to toxic effects of certain chemicals can be early warning indicators of decline and extinction, community function, and ecosystem services. Population level changes can occur because of individual toxic effects such as changes in reproduction (*e.g*., sperm motility, delayed hatch, and smaller brood size), as well as reduced fitness caused by nutrient substitution. In addition, changes in community structure, such as increased or decreased predation or shifts in prey availability, may have adverse effects on populations. The few studies of population level effects from tire particles and associated chemical additives include laboratory toxicity studies demonstrating effects on reproduction that may affect wild populations and ecosystems (Table 6). In general, studies that spike tire particles into sediments appear to show fewer adverse effects than those using tire particles in water. However, the method by which tire wear particles are produced for toxicity studies may make a large impact on their environmental relevance and toxicity. Most studies use pieces of a single tire to cryomill particles, which results in tire fragments that may look and act quite differently from particles generated by tire use. Panko et al. (2013) is one of the few studies where the environmentally relevant abrasion of tires was mimicked to produce the tire particles used for toxicity testing. Whether how tire particles are produced makes a significant difference in their toxicity is an important data gap for interpreting most tire particle toxicity studies to date.

Both tire particles and associated leachates can cause shifts in microbial marine communities that may affect nitrogen metabolism in marine sediments (Liu et al., 2022; Wik et al., 2009; Yang et al., 2022). While there are few studies of community level effects resulting from direct tire particle exposure, there are data on some of the chemical additives associated with tires, including metals. For example, changes in community tolerance to increased Zn levels in sediments (>124 mg/Kg dw) has been demonstrated for microbial communities (van Beelen, 2003), and with Zn, Cd, and Cu for river periphyton (Blanck, 2002). Effects of Ni on diatom communities were reported at 1000 μg/L (Ivorra et al., 2002) and for freshwater benthic communities at ~500 mg/Kg depending upon the acid volatile sulfide and total organic carbon concentrations of the impacted sediments (Nguyen et al., 2011). Schlekat et al. (2016) summarized studies on benthic freshwater communities and determined that NOEC Ni levels ranged between 100 and 230 mg Ni/Kg dw. Among freshwater nematode communities, shifts in species composition start at 13mg/Kg to 19mg/Kgdw and porewater concentrations of 20μg/L to 30μg/L (Louati et al., 2013). PAHs have also shown community level effects. Alpha diversity and evenness of sediment prokaryotic communities changed after pyrene exposure at 250 mg/Kg (Ding et al., 2017). Sediment meiofauna community and microbial processes were altered at PAH concentrations of 130 to 1300 μg/Kg dw sediment (Lindgren et al., 2014) and the meiofauna community was disrupted by environmentally occurring PAH mixtures of 45 mg/Kg, 30 mg/Kg, and 25 mg/Kg of dry sediment for fluoranthene, pyrene, and phenanthrene, respectively (Louati et al., 2013). Other tire additives such as BPA also can cause microbial community changes; effects of BPA were seen at concentrations of 180 mg BPA/Kg dw sediment (Yang et al., 2014).

While most published studies on adverse effects caused by tire particles, metals, hydrocarbons, and associated chemical additives are related to the aquatic environment, terrestrial endpoints may also be impacted. For example, Selonen et al. (2021) found a significant reduction in the reproduction of the springtail *Folsomia candida* in soils spiked with tire rubber particles at concentrations up to 1.5 % of soil dry weight. In contrast, at the same concentration, no significant effects were found on reproduction of the worm *Enchytraeus crypticus* (Selonen et al., 2021) or of *F. candida* exposed to tire particles from bicycles, cars, and e-scooters (Kim et al., 2022).

Reported adverse effects of tire particles on soil invertebrates are highly variable for oligochaetes, annelids, isopods, arthropods, and nematodes, but suggest that tire wear particles could adversely affect soil invertebrates at concentrations found at roadsides in the environment (Ding et al., 2020; Pochron et al., 2017, 2018; Selonen et al., 2021; Zhao et al., 2011). In addition, a 15 % treatment of crumb rubber reduced soil bulk density by 28 % and soil moisture by 6 % (Zhao et al. (2011). While the concentration of tire particles found to have an effect in this study was relatively high, it is likely representative of current or future soil tire content given the constant generation of tire particles on roadways and a lack of mitigation strategies. At the ecosystem level, whole tires collect water, serving as habitat and shelter for pests such as mosquitoes and bacteria or disease-carrying rodents (Torretta et al., 2015). MNPs, under which tire particles can be categorized, can influence the structure and function of ecosystems (Rillig and Lehmann, 2020). Therefore, tire particles should be considered as an agent of change at the ecosystem level. Tire particles may serve as substrata for colonization by microbes (Torretta et al., 2015). Ecosystem level effects from metals, as well as other impacts caused by environmental parameters (*e.g*., ionic strength, pH) may also result when tire wear particles are present. For example, in a laboratory study of a marine benthic system from the Baltic Sea, Sundelin and Elmgren (1991) reported adverse impacts to nematode abundance, total meiofauna abundance and biomass, non-nematode biomass, ostracod abundance, and Turbellarian abundance resulting from exposure to Cd. In contaminated sediments on the northern Australian coast, based on genomic analysis, the presence of several metals (*e.g*., Al, V, Zn, Cu, Ga, Pb, Cd, and As) negatively impacted Archaea and bacterial (Cornall et al., 2016). In another field study from Australia, invertebrate predator ratios and taxa richness in freshwater streams were negatively affected by Cu and Zn (Liess et al., 2017). Versteeg et al. (1999) demonstrated the impacts of metals to ecosystems ranging from disruptions to community structure, colonization, and productivity, to reductions in taxa richness, growth, and survival. Their synthesis demonstrated that cationic metals such as those leached from tire particles could cause adverse effects to ecosystems. However, no ecosystem level studies have been performed on tire particles themselves in aquatic systems. Both aquatic and terrestrial ecosystem effects of tire particles and associated chemical additives are, to date, poorly studied and represent a large data gap in our understanding of the adverse ecological effects of tires.

In addition to tire particles and associated chemicals, environmental factors such as elevated ion concentrations from road salt (*e.g*., Ca^2+^, Na^+^, Cl^−^) can cause synergistic effects (Kaushal et al., 2020). For example, Klauschies and Isanta-Navarro (2022) found that, at environmentally relevant concentrations, the joint action of salt and 6PPD-quinone had an antagonistic, negative effect on the reproduction and survival of freshwater rotifers. Further, the presence of tire debris can change soil pH, potentially influencing availability of nutrients and toxic metals (Smolders and Degryse, 2002; Councell et al., 2004). MNPs can contribute to the formation of anthropogenic soils and carbon stabilization by influencing bulk density, soil aggregate size, porosity, and water holding capacity (Rillig et al., 2021) which, in turn, limits growth and survival of plants (Bollman et al., 2019). While the effect of nanoplastics on terrestrial ecosystems is largely unknown, nano-sized particles may be taken up by plants themselves (Rillig et al., 2021). Leifheit et al. (2022) showed experimentally that tire abrasion particles reduced aboveground and belowground plant biomass, increased soil respiration and pH, and affected litter decomposition even at their lowest treatment concentrations of 10 mg/g of tire particles (average size of 125 μm; range of 34–265 μm). Castan et al. (2023) showed that lettuce plants exposed to tire-derived compounds including diphenylguanidine, hexamethoxymethylmelamine, benzothiazole, 6PPD, and 6PPD-quinone at concentrations of 1 mg/L were readily taken up and metabolized by the plants and, furthermore, leaching from tire particles continually resupplied the metabolized compounds to the lettuce leaves. The role of tire wear particles as stimulators or inhibitors of biogeochemical and ecosystem functions in urban systems and the potential for ecological and human health risks warrants further consideration and investigation.

Studies show that tire particles can influence C, N, and P cycles directly and indirectly by serving as a source for these elements in food webs (Bucci et al., 2020; Xu et al., 2020). The high C:N ratios of tires and aromatic hydrocarbons stimulate immobilization of N in soils by microbes (Rillig et al., 2021). Conversely, plastics are of lower quality and lability than simpler natural and anthropogenic carbohydrates. Consequently, in the presence of lower litter quality, microbes seek N in the soil organic matter to process the C from the litter, thus inducing excess C loss from soils (Averill and Waring, 2018). Such processes may vary among ecosystem types or along successional gradients (Mayer, 2008). Future studies could investigate the effects of tire particles on decomposition rates because toxic cationic metals and toxic organic chemicals can inhibit microbial and biogeochemical processes and rates (Baensch-Baltruschat et al., 2020; Rødland et al., 2022). Tire particles can release nonpolar hydrocarbons, which may lead to accumulation of C in upper soil horizons due to their limited mobility (*sensu* Lajtha et al., 2005). Ingestion of plastic particles like tire particles may cause lesions and reduce reproductive success in earthworms and affect other soil arthropods (Büks and Kaupenjohann, 2020), though the full range of effects of tire particles on soil macrofauna are unknown. Tires and tire particles are also rich in organic N and P, but less is known regarding their bioavailability compared to C.

### 4.7. Human health effects

Some tire chemicals are known toxicants to terrestrial organisms and humans (*e.g*., many metals, PAHs), but the potential toxic effects of many tire-derived chemicals on terrestrial organisms remain unknown. For humans, oral and dermal exposure to tire particles is expected to be minimal compared to inhalation exposure (Kreider et al., 2020). While the larger fraction of particles is primarily in the non-respirable size range (5 μm–220 μm, mode approximately 75 um; Kreider et al., 2010), smaller sized tire particles (PM10 and PM2.5) contribute to particulate air pollution that can get deep into the lungs and into the bloodstream. Du et al. (2022) found 6PPD and 6PPD-quinone in the urine of children and adults including pregnant women in China, showing that tire-derived chemicals can get into the bloodstream, presumably from inhalation exposure to tire particles. While tires are a major source, other rubber materials such as belts hoses and cables also may emit 6PPD and 6PPD-quinone (Bohara et al., 2024; Cao et al., 2022), and facilities such as sports fields constructed wth tire crumb may be exposure points for humans (Skoczyńska et al., 2021). Mice exposed orally to 6PPD and 6PPD-quinone showed signs of hepatotoxicity from bioaccumulation in liver tissue along with disorders of lipid metabolism and inflammatory immune response due to gene upregulation, suggesting potential health risks to mammals (Fang et al., 2023).

The toxicity of tire particles has been investigated in various *in vitro* studies, which have consistently shown DNA damage and inflammatory effects, while *in vivo* studies using rats and mice have found inflammatory response and pulmonary toxicity from exposure to tire particles (Baensch-Baltruschat et al., 2020). Based on a mouse model and epigenic markers, inhalation of tire wear particles in the size range of <1 μm (hydrodynamic diameter distributed at 100 nm) caused significant pulmonary fibrotic injury (Li et al., 2022). Kreider et al. (2020) developed a species- and time- adjusted no-observed-adverse-effect-concentration (NOAEC) for respirable tire and road wear particles of 55 μg/m^3^ based on cardiopulmonary effects. Comparing this NOAEC to predicted exposure concentrations, they concluded that tire and road wear particles present a low risk to human health, especially when compared to other air pollution sources like diesel exhaust. However, this analysis is based on limited data and did not consider particularly sensitive or highly exposed population groups or other effects such as development of asthma, cancer, or reproductive toxicity.

## 5. Managing and remediating pollution from tires

**Fig. 4.**
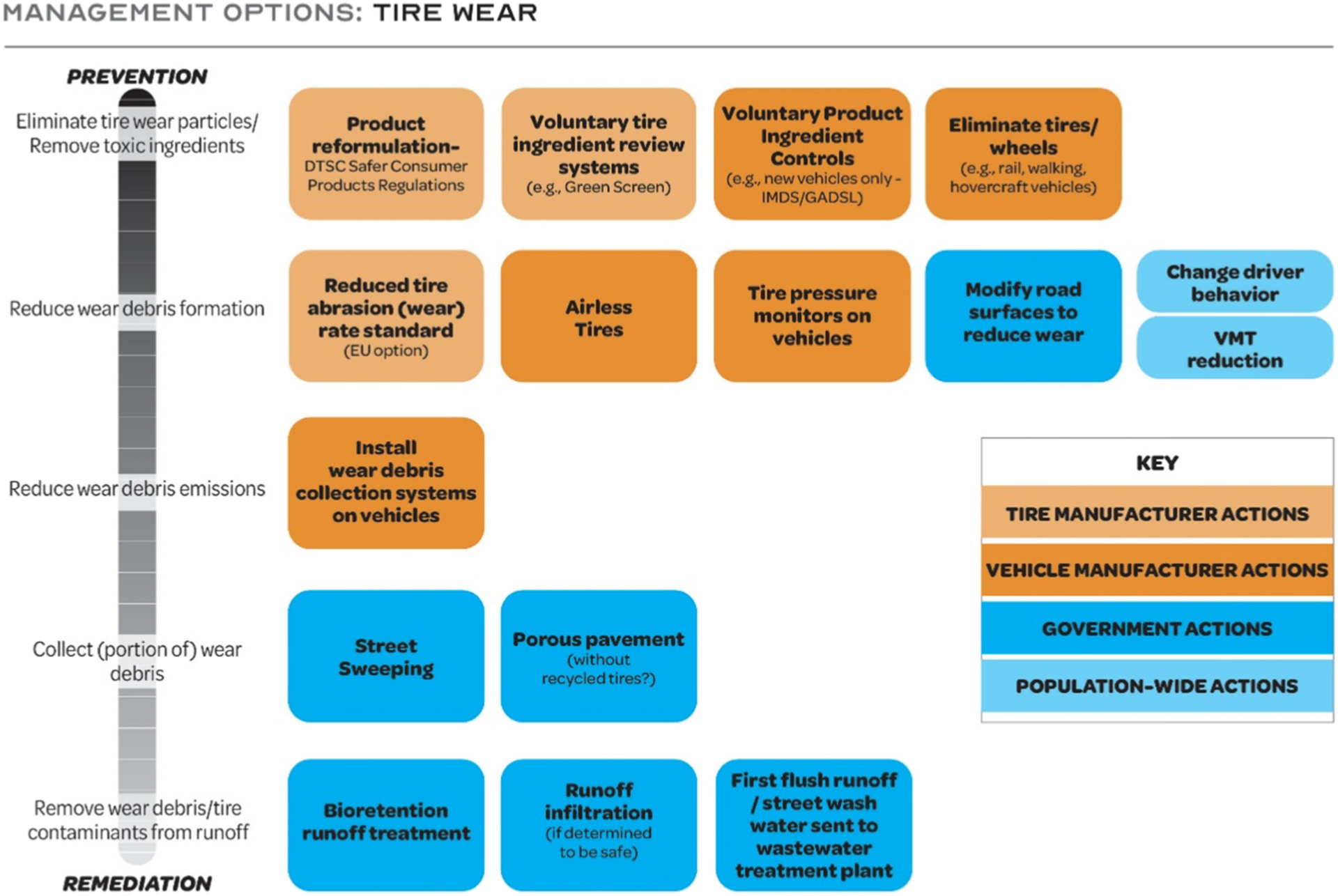
Management options for tire wear particle pollution, framed on a scale from preventative (reducing use and release) to remedial (collection and removal) measures. Options are color-coded by who would be responsible for implementation, with tire manufacturers in light orange, vehicle manufacturers in dark orange, government in dark blue, and community-wide in light blue. Reprinted from Moran et al. (2021).

Currently, few remediation and management technologies are focused on tire particles or related chemicals (Schmaltz et al., 2020). Potential mitigation opportunities for uncontrolled environmental release of tire particles and chemicals include actions by four sectors: tire manufacturers, vehicle manufacturers, government, and the general population. Approaches for mitigating tire particle and related chemical pollution range from prevention (*e.g*., reformulating tires to remove toxic ingredients and reducing tire wear debris formation and emissions) to collection and treatment (*e.g*., capturing tire particles or tire-related chemicals after dispersal into the environment) (Fig. 4). Efforts to reduce overall vehicle kilometers traveled by promoting or making more accessible alternative forms of travel or public transportation could reduce tire emissions. Addressing tire particles and tire-related chemicals will require a holistic approach to address both the tire microplastics and the chemicals they contain because multiple entities and agencies typically manage different components of the tire life cycle. In an extensive review, Baensch-Baltruschat et al. (2020) summarized possible mitigation approaches for reducing tire wear particles emitted to the environment including: modernized installation and retrofitting of stormwater treatment systems, improved runoff treatment systems on rural roads and highways including disposal of sediment, incineration of sewage sludge containing road runoff sediments instead of spreading on agricultural areas or natural soil, regular maintenance of roads, lighter weight vehicles, reduced traffic and speed limits, and optimization of tire materials to improve wear resistance. Autonomous cars may improve emissions because they could be operated to brake and accelerate less often. Treating road runoff, particularly at pollution hotspots such as tunnels, was also suggested (Baensch-Baltruschat et al., 2020). However, because tire particles follow various paths, estimated at proportions of 49 % for runoff, 49 % for soil and 2 % for aerial transport (Unice et al., 2019), multiple mitigation approaches along pathways are necessary for effective control.

### 5.1. Reformulation

Ingredient assessments (*e.g*., as implemented by the California Department of Toxic Substances Control; California DTSC, 2021) could provide information to inform potential tire tread reformulation to address aquatic toxicity associated with existing formulations. Existing chemical hazard evaluation tools like Green Screen for Safer Chemicals or the USEPA Hazard Comparison tool (*vide infra* Vegosen and Martin, 2020) might provide a framework for such evaluations (Clean Production Action, 2021). The international Global Automotive Declarable Substance List (GADSL) provides a list of chemicals of interest due to existing or anticipated regulation, human or environmental hazard, or association with functional problems in vehicle parts (Technology, 2021; Global Automotive Stakeholders Group, 2021). The GADSL provides vehicle manufacturers with data on chemical ingredients for use in tires that could be considered in reformulations to address potential toxicity concerns. Tire manufacturers report seeking sustainable materials to develop next generation tires (Amick, 2018). However, limitations to this search include manufacturer competition, proprietary information protection, and technological barriers to formulating tires with desired wear and handling characteristics.

### 5.2. Reduced tire wear debris formation

Multiple organizations are working to redesign and reformulate tires to reduce wear debris formation, potentially involving regulatory standards (*e.g*., European TRWP Platform, 2019; Hann et al., 2018; Piscitello et al., 2021; European Tyre and Rubber Manufacturer’s Association, 2021; Verschoor et al., 2016). Between 2015 and 2021, tire manufacturer Michelin claims to have reduced its overall tire wear mass emissions by 5 % (de Peufeilhoux et al., 2021), which may explain why a comparison test of tires from major European manufacturers showed Michelin had the lowest tire wear rates (Silvestro, 2022). In 2022, the European Commission proposed to regulate tire wear rates (European Commission, 2022).

Maintaining tire pressure, either through airless tires (a technology already entering the market) or tire pressure monitors, has potential to reduce wear rates by an estimated 14 % (Verschoor and de Valk, 2017). Improvements in tire pressure monitoring systems, such as tire-specific warnings and a more sensitive trigger for the warning could maximize reductions. Resurfacing roads and increasing road maintenance to create smoother surfaces may reduce tire wear rates (Alexandrova et al., 2007; Kole et al., 2017) but may entail high implementation costs likely to limit the widespread implementation of such measures. Changing driver behavior (*e.g*., reducing speed, hard cornering, and braking) could reduce wear debris formation (Kole et al., 2017). However, public education is only effective when costs, benefits, and social norms support changed behaviors. Future vehicle automation could provide a more realistic path for behavior-related tire emissions reductions. Also, roads could be designed to reduce travel speeds that would, in turn, reduce tire wear though, road reconstruction or retrofit may be prohibitively costly and would have its own impacts.

### 5.3. Reduced tire wear debris emissions

Capturing pollutant emissions at the source typically is more cost-effective than removal from the environment after dispersal. Three companies are patenting systems to collect tire wear debris on vehicles (The Tyre Collective, Nexen Tire, and GelbKo Environmental Solutions). The effectiveness of such systems will depend, in part, on capturing particle sizes with the greatest potential to release chemicals or to harm aquatic organisms. Understanding which vehicle classes (*e.g*., cars, heavy duty trucks) emit the greatest total quantity of tire wear debris would inform priorities for installation of such systems.

### 5.4. Collection and treatment of dispersed tire particles

Collecting tire particles in the environment poses significant challenges. Existing street sweeping programs are episodic and do not address all impervious surfaces where tire particles deposit (*e.g*., sidewalks, driveways, and rooftops). Mechanical street sweepers are only moderately efficient at collecting particles in the size range of tire wear debris and may release microplastics from the sweeping brushes themselves (Amato et al., 2010; Piscitello et al., 2021; Selbig and Bannerman, 2007). Particles may also be collected by clearing deposition locations in the runoff transport system like roadside ditches, catch basins, and storm drainpipes before they are washed into surface water (Navickis-Brasch et al., 2022). Larger tire fragments, such as those from failed retread tires, could be manually collected at roadsides prior to decomposition. Currently, the portion of all tire wear particles collected cannot be reliably estimated from available data (Klöckner et al., 2020; Polukarova et al., 2020; Sieber et al., 2020).

Although it increases tire wear, porous pavement may reduce tire particle wash off by retaining a significant fraction (as much as 40 %) of emitted tire wear particles (Stanard et al., 2007; Verschoor et al., 2016). It is unknown if retained particles subsequently wash away. Furthermore, retained tire particles could continue to release tire-related chemicals into the environment over time. While some countries (*e.g*., Netherlands) make extensive use of porous pavement (Verschoor et al., 2016), such pavement for automobile traffic is uncommon in the US (Caltrans, 2021; Passeport et al., 2013), may not last as long as conventional pavement, and it may be cost prohibitive to replace existing pavement.

### 5.5. Treatment to remove tire particles and chemicals from water

Because urban and road stormwater is a significant pathway for tire particle transport (Moran et al., 2021; Werbowski et al., 2021), prioritizing stormwater runoff is key to capturing tire particles and chemicals. Urban runoff treatment systems or combinations of these systems (“best management practices” or BMPs) have potential to reduce tire particles and chemicals released to waterways. Examples of urban runoff BMPs include “green infrastructure” such as vegetative buffers along water bodies (Mayer et al., 2007), rain gardens, grassed swales, retention structures such as ponds, sand filters and filter strips, wetlands, and infiltration features such as trenches and basins (Martins et al., 2015; Galella et al., 2023) and “gray infrastructure” such as hydrodynamic separators. Studies have demonstrated that stormwater bioretention facilities can effectively remove inorganic and organic pollutants from stormwater, including removal of tire wear particles (Gilbreath et al., 2019, Smyth et al., 2021, Mengistu et al., 2022, and McIntyre et al., 2023). The effectiveness of these facilities varies depending on a range of factors, including their design and maintenance. Assessing stormwater treatment effectiveness is a significant challenge when not all of the constituent chemicals in tires are currently known. The most promising stormwater treatment systems for tire particles and chemicals are rain gardens (bioretention cells) and similar soil-based pollutant capture and infiltration systems. Rain gardens (engineered depression features of soil, organic matter, and vegetation) are known to be highly effective at capturing pollutants from stormwater including metals, salts, and nutrients (*e.g*., Passeport et al., 2013; Gilbreath et al., 2019; Lange et al., 2021; Laws et al., 2011; McIntyre et al., 2015; Smyth et al., 2021; Galella et al., 2023; Kaushal et al., 2022; Maas et al., 2023). Both Gilbreath et al. (2019) and Werbowski et al. (2021) found that rain gardens were effective at reducing microplastic concentrations in water (>90 %). Removal was greatest for particles 3.5–5.0 mm but lesser for particles <0.5 mm, yet reportedly 100 % for rubbery fragments which were assumed to be primarily from tires (Werbowski et al., 2021). McIntyre et al. (2015) observed high efficacy of engineered soil matrixes typically used in bioretention systems for attenuating the toxic leachates from tires responsible for coho salmon mortality in the Pacific Northwest USA. Optimizing filtration systems will be partially a function of the capture system design, including the soil or aggregate matrix selected for filtration (Bollman et al., 2019; Zhang et al., 2021) and the balance of organic matter, clay, other potential additives (*e.g*., biochar), and sand and trade-offs with other microbial and biogeochemical processes such as nutrient transformation (*e.g*., denitrification) where organic matter type and proportion can unintentionally contribute to metal or phosphorus mobilization (Duan et al., 2019).

A few green infrastructure designs have shown promise for some removal of other microplastic types (Stang et al., 2022). Significant tire particle and chemical removal would only be expected in systems that provide significant removal of fine solids or dissolved pollutants, suggesting soil and wetland-based green infrastructure would likely outperform gray infrastructure and many retention pond designs (Clary et al., 2020; Wagner et al., 2018; Navickis-Brasch et al., 2022). Infiltration-based runoff treatment systems could allow soluble tire-related chemicals to pass into groundwater, raising questions about the safety implications for groundwater aquifers that are current or future drinking water sources. Furthermore, biofiltration-based stormwater features may, in the long term, be a source or a sink of tire-derived chemicals like 6PPD-quinone, and metals or base cations depending on hydrology, soils, or other factors such as redox potential, pH, and salinity (Galella et al., 2021; Galella et al., 2023; Navickis-Brasch et al., 2022). Though not widely implemented in the US, some municipalities direct “first flush” runoff from select road segments into the municipal wastewater collection system that flows to wastewater treatment plants, which likely remove a significant portion of tire particles (Wagner et al., 2018). Deliberate, pre-rain event washing to reduce pollutants on road surfaces has also been suggested (Kreiger Jr., 1994).

Emphasis on management and mitigation of tire-derived chemicals has recently been focused on 6PPD due to its connection with coho salmon mortality in the Pacific Northwest USA. For example, the Washington State Department of Ecology produced a comprehensive document on BMPs for stormwater treatment of tire contaminants (Navickis-Brasch et al., 2022) which concluded that capture and attenuation methods will depend upon the characteristics of 6PPD and 6PPD-quinone, which appear to bind to soil and organic matter, remain undissolved and buoyant, and may have long half-lives (6PPD has a half-life of *ca*. 5 h, while 6PPD-quinone’s half-life may be several days; DTSC 2022 Product Chemical Profile). Furthermore, 6PPD will continue to leach from tire particles and continue to transform to 6PPD-quinone in the presence of ozone or oxygen. Therefore, retention methods will need to be effective over the lifespan of release of the particles, depending upon environmental and physical conditions, including temperature, pH, presence of metals, redox, salinity, *etc*. BMPs with the highest potential to reduce 6PPD and 6PPD-quinone are infiltration, dispersion, and biofiltration combined with sorption, especially those containing soil media or compost (Navickis-Brasch et al., 2022). In a controlled experiment, salmon survived otherwise deadly, untreated stormwater when the stormwater was filtered through a bioretention soil mixture containing 60 % sand, 15 % compost, 15 % shredded bark, and 10 % drinking water treatment residuals (McIntyre et al., 2015). Similarly, Spromberg et al. (2016) successfully prevented mortality of coho salmon by treating stormwater through biofiltration media. In a more recent study, McIntyre et al. (2023) found that experimental bioretention filtration through a mixture of sand and compost (60:40 by volume) overlying a gravel drainage layer and covered with wood bark mulch, removed bacteria, dissolved metals, and polycyclic aromatic hydrocarbons, and likely also effective removal of 6PPD, thereby preventing all mortality of adult coho salmon and alevin, or newly hatched salmon, exposed to stormwater. In another study, stormwater bioretention cells designed to redirect stormwater away from receiving waters provided a 10-fold reduction in 6PPD-quinone mass loadings to receiving waters (Rodgers et al., 2023). The mechanism of action and longevity of such treatments are unknown (Navickis-Brasch et al., 2022). Identifying optimal soil media mixtures for bioretention and other characteristics is a critical need (Bollman et al., 2019). Designing effective bioretention systems will depend upon tailoring their construction to specific urban landscapes and/or retrofitting existing systems (USEPA, 2023). Bioremediation facilities require inspection and maintenance to ensure adequate performance over multiple years. Occasionally material may need to be removed, tested for contaminants, and disposed of properly (USEPA, 2016).

## 6. Tire recycling, reuse, and disposal

**Fig. 5.**
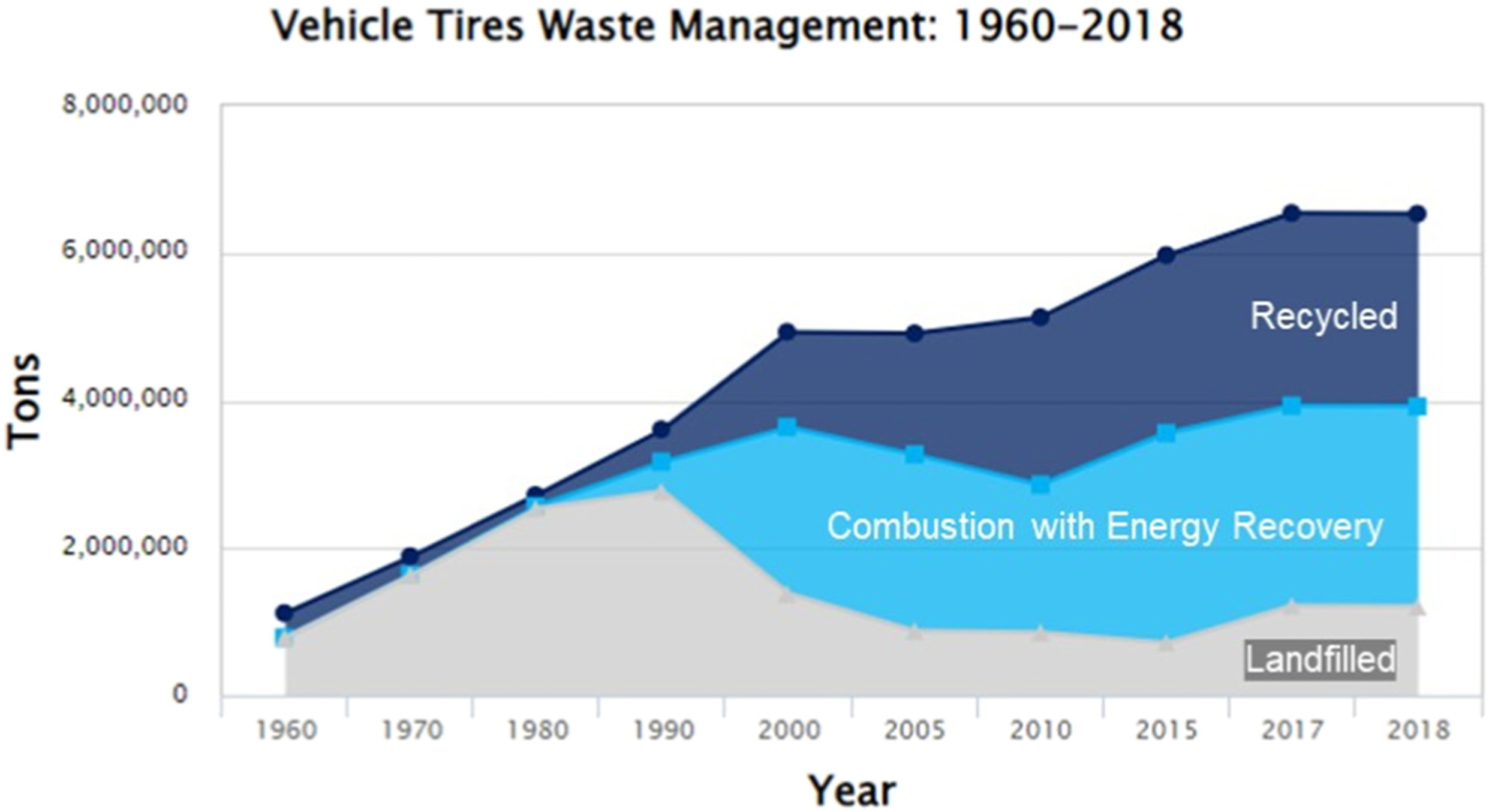
Vehicle tires waste management: 1960–2018. Weights in US tons. Reprinted from https://www.epa.gov/facts-and-figures-about-materials-waste-and-recycling/durable-goods-product-specific-data#VehicleTires.

Waste (scrap) tires or “end of life tires” represent a significant volume of the international solid waste stream (Grammelis et al., 2021). In 2019, over 4.05 million metric tons of scrap tires were generated in the US compared to 3.56 million metric tons in the EU (Table 2). Brazil produced 473,000 tons of waste tires in 2015 alone (Machin et al., 2017). While the volume of waste tires over time has increased since 1960, fewer tires are landfilled, and more are either recycled or combusted as an energy source (USEPA, 2022a, 2022b; Fig. 5). Tiered waste management hierarchy starting with pollution prevention is usually more cost effective than control or treatment and disposal of waste products (USEPA, 2023). The most effective pollution prevention approaches involve creating a circular economy where product-related waste becomes the source material for another generation of the same product. Tires pose a challenge for this hierarchy, as they wear away during their lifetimes, losing about 10 % of their mass in the form of tire wear particles emitted into the environment (USTMA, 2021). While such emission and subsequent dispersion prevent a fully closed-loop life cycle, new prevention options are in development, some of which could feed into a future circular economy (*e.g*., on-vehicle collection of tire wear particles and end-of-life tire rubber separation and devulcanization; The Tyre Collective, 2021; Markl and Lackner, 2020).

International, national, and US state regulations prohibit the disposal of most tires in landfills (*e.g*., Dabic-Miletic et al., 2021; Dajić et al., 2016). While there are no US federal programs to facilitate recycling or reuse of tires, 48 states have regulations that control tire disposal. Similarly, there are no special EU standards for used tires and countries may choose a management system (Torretta et al., 2015). Some US states have developed sustainable statewide market infrastructure for tire-derived products such as California’s Department of Resource Recycling and Recovery (CalRecycle, 2019, 2020, 2021).

In the EU, only about 4 % of tires are deposited in landfills while 38 % are used for energy, 40 % are recycled, and 18 % are reused in some way (Torretta et al., 2015). Stockpiled, landfilled, and illegally dumped tires can be a source of pollution to groundwaters (Skoczyńska et al., 2021) and can hold water, making them notorious breeding sites for disease-carrying mosquitoes and bacteria (Torretta et al., 2015). In the EU, 2 million tons of tires are stockpiled or abandoned illegally (Torretta et al., 2015). In the US, stockpiled tires have declined steadily from 1 billion tires in 1990 to about 56 million tons in 2019, with stockpile concentrations in a few states such as Texas and Colorado (USTMA, 2020). Only six states do not have storage and disposal regulations, 11 allow whole tires in landfills, and 37 states allow cut or shredded tires to be deposited in landfills (USTMA, 2020), while about half of all states have an active stockpile cleanup program. Stockpiles are susceptible to tire fires either inadvertently (*e.g*., wildfires), through arson, or as a disposal method which releases smoke containing particles and toxic pollutants such as carbon monoxide (CO), cyanide, sulfur dioxide, butadiene, and styrene (Singh et al., 2015). Such fires may burn out of control for many days or months, consuming millions of tires (Kordoghli et al., 2014; Singh et al., 2015). Assessment of an uncontrolled fire at a landfill showed emission of high concentrations of CO, CO_2_, SO_2_, fine particulate (PM2.5), and PAHs including nitrogen heteroatoms (azaarenes) and picene, which are also found in coal combustion (Downard et al., 2015).

In 2019, 75.6 % of scrap tires in the US and 95.6 % in the EU were processed in some manner that kept them out of landfills (USTMA, 2020; European Tyre and Rubber Manufacturer’s Association, 2021). The three major categories of tire disposition other than deposit in landfills are energy production, recycling, and civil engineering. Whole tires are often used as fuel to generate electricity or for manufacturing processes (USTMA, 2020; Ruwona et al., 2019). For example, in the EU, 40 % of tires are burnt, mostly in cement kilns (Piotrowska et al., 2019). In the US, 100 million tires (1.4 million tons) were burned as fuel for cement kilns, paper industry, and industrial boilers (USTMA, 2020). Combusting tires for fuel is primarily employed in developed countries and mainly for cement manufacturing which requires significant energy consumption (Grammelis et al., 2021). The energy value of tires for fuel is equivalent to oil but produces fewer NOx emissions (Grammelis et al., 2021). However, the combustion of waste tires produces atmospheric pollutants such as dioxins, dibenzofurans, NO_x_, SO_x_, and heavy metals remain in the ash such as Zn, Mn, Cr, Pb which present disposal issues (Chen et al., 2022). However, Cu can be removed from industrial wastewater using tire ash as an absorbant (Mousavi et al., 2010). Tire ash is also often incorporated into asphalt and Portland cement (Al-Akhras and Smadi, 2004). Tires may also be pyrolyzed or gasified which provides higher energy efficiencies, produces less gaseous pollutants than combustion and enables recovery of by-products like Zn or char for activated carbon production and carbon black, though there are limits to the utility of this type of char due to impurities (Grammelis et al., 2021; Chen et al., 2022). While the gas, liquid, and solid (char) products from pyrolysis of tires are useful for energy production, carbon black, graphene, and others, further research and technologies are needed for tire pyrolysis to be a sustainable process for disposing tires at an industrial and economic scale (Kommineni et al., 2018; Gao et al., 2022).

Recycling tires yields rubber, steel, textile fibers, and carbon black (Fazli and Rodrigue, 2020). Rubber may be reused as fillers, in asphalt and road pavements, and in manufacturing of new tires, while recycled steel can replace anthracite and coke, and textile fibers which amount to about 5 % of the total mass of the tire, may be recycled or incorporated into products such as sound absorbing materials or mixed into plastics or cement for reinforcement, or as a geopolymer to reinforce soils (Dong et al., 2021; Grammelis et al., 2021). The second largest use of scrap tires in the US is for ground rubber. About 66 million tires (1 billion tons) are used in asphalt, sports surfaces, artificial mulch, and for other molded manufactured goods (USTMA, 2020). Europe disposes of over 51 % of scrap tires, including significant amounts of granulated rubber (ETRMA 2021). A smaller but still significant use of scrap tires is for civil engineering purposes (Grammelis et al., 2021), including backfilling and road construction (Table 2). Tires used as fuel can release potent carcinogenic particulates to the atmosphere including HCN, NOx, SOx, CO, and CO_2_ (Glushankova et al., 2019). NOx and SOx emissions occur when tires are used to fuel combustion in cement kilns (Nakomcic-Smaragdakis et al., 2016). If facilities are designed properly, emissions from use of tires as fuel are unlikely to be greater than those from conventional fuels such as coal, coke, or wood (USEPA, 1997).

About 226,000 tons of tires were used in civil engineering projects in the US in 2019 (USTMA, 2020). Tire rubber pieces (tire-derived aggregate, crumb rubber) are sometimes used in stormwater capture and treatment systems (*e.g*., bioretention) or as bottom fill for roadside drains (CalRecycle, 2016; Deng et al., 2016). Crumb rubber may remove some pollutants (*e.g*., metals) from runoff (Deng et al., 2016). However, tire microplastics and tire-related chemicals may be released into urban runoff or groundwater (Moran et al., 2021) as may be the case for whole tires used in engineering such as for road fill and construction (Torretta et al., 2015). Shredded tires are sometimes used as landfill liners, which may pose risks from groundwater seepage and, in some cases, inadvertent burning (Downard et al., 2015). For example, tire-derived aggregates in stormwater management features can leach Fe, Cu, and Zn at levels above chronic criteria for aquatic biota (Singh et al., 2023).

Artificial turf installations use infill composed of crumb (ground) rubber microplastics created by cryomilling or granulation of whole used tires into microplastics 0.25–4 mm in size (USEPA, 2019). Turf installations can represent significant inputs of tire rubber to the environment with over 11,000 synthetic turf fields across the US (Perkins et al., 2019). In 2017, there were over 60,000 synthetic turf football fields and pitches in the EU, not including children’s playgrounds or indoor recreation facilities (Skoczyńska et al., 2021). While crumb rubber loss estimates vary (Hann et al., 2018; Kole et al., 2017), the wide use of crumb rubber in the construction of artificial turf on football fields has prompted concerns about potential health risks of hazardous substances present in recycled rubber and a possible link to lymphoma and leukemia risk among sports players (Skoczyńska et al., 2021). These concerns, in part, led to the development of the “Federal Research Action Plan on Recycled Tire Crumb Used on Playing Fields and Playgrounds” (USEPA, 2019). Human exposure to toxics from crumb rubber may be *via* inhalation, ingestion, or contact depending upon specific use of the playing surface as well as environmental factors such as temperature; for example, heavy metals may be inadvertently ingested from particles, while PAHs are more likely to be inhaled through off-gassing of turf during high temperatures (Armada et al., 2023; Perkins et al., 2019; Zhang et al., 2008). Chemical leaching from crumb rubber also has potential negative effects on aquatic biota (Halsband et al., 2020).

Road surfaces may contain tire crumb, either as a component of asphalt pavement (rubberized asphalt) or as a component of a surface coating (chip seal or slurry seal). In California, USA, while tire crumb rubber composes only a small fraction (about 1.6 %) of the final pavement by weight, each metric ton of pavement contains about 16 kg of crumb rubber (Caltrans, 2020). Particles released when rubberized asphalt, chip seal, and rubberized seal coats wear away are primarily rock and non-rubbery asphalt/tire crumb blends, though volumes of emissions from rubberized asphalt are currently unknown and questions remain on potential impacts to air and water pollution (Buttlar, 2021). Lokesh et al. (2023) found that rubberized asphalt concrete mixtures both sorbed and released 6PPD-q in leachates over time.

Overall, the fate of end-of-life tires is varied, and each end use has different environmental impacts, ranging from the virtual elimination from the waste stream (*e.g*., incineration, incorporation into cement) to a continued presence (*e.g*., asphalt, tire crumb) that requires additional disposal considerations (Sienkiewicz et al., 2012; Fazli and Rodrigue, 2020).

## 7. Regulation and legislation

Regulations and policies can reduce emissions and negative impacts from tire particles (Trudsø et al., 2022; https://unece.org/transport/vehicle-regulations). Policy instruments directed at multiple levels to address risks across the tire life cycle have the potential to affect the production, dispersal, or characteristics of tire pollutants (Johannesson and Lithner, 2022), including:

- tires (degradability, type of tire, wear propensity, tire pressure, hazardous substances).
- vehicles (weight, number of axles, engine power, acceleration, wheel alignment).
- road layout (engineering designs that encourage high speeds or sudden braking and acceleration).
- road operation and characteristics (snow clearing, road cleaning, road surfacing material, management of stormwater, presence of roadside vegetation).
- traffic patterns (physical planning, public transport, taxes, parking fees).

Various countries and jurisdictions are using guidance and regulatory tools to address management of tire pollutants. For example, legislation in Europe addressing tires as hazardous waste dates to 1975 while other legislation over years has addressed disposal of tires, shipment of waste, and “polluter pays” principles (Grammelis et al., 2021). In the EU, there are three options for managing end-of-life tires: a) Extended Producer Responsibility where the producer is fully or partially responsible for an end-of-life product; b) Free Market System where countries operate under specific contract agreements and often report to respective regulatory agencies (*e.g*. Austria, Switzerland, Germany, and the UK; c) Tax System where a tax is imposed upon tire producers, which is indirectly paid by the consumer (*e.g*. Croatia and Denmark).

The World Forum for Harmonization of Vehicle Regulations (WP.29), part of the United Nations Economic Commission for Europe, issued global technical guidance for regulation of tires (Global Technical Regulation no.16; UNECE, 2014) with environmental and performance-oriented test requirements. These regulations encompass tire wear indicators but not tire wear particles or emissions of other tire-derived chemicals. World Forum regulations are not enforceable. Rather, contracting parties agree to adopt regulations and enforce respectively (UNECE, 2014). Under-inflated tires release more wear particles than those properly inflated. The European Parliament and Council required all new passenger vehicles in the European Union to be fitted with tire pressure monitoring systems beginning in 2014. The US, China, Taiwan, and South Korea (van Zyl et al., 2013) also mandate tire pressure monitoring systems. The Regulation of the European Parliament and Council (EU 2020/740) on tire labeling includes provisions for adding parameters on tire abrasion and when tires need to be replaced due to wear (European Council, 2020). Currently, the only European Union restriction on chemical content of tires is a PAH limit of 1 mg/kg for benzo[*a*]pyrene (BaP) and 10 mg/kg for the sum of PAHs. The Austrian National Road Administration requires that road runoff is treated when the annual daily traffic reaches 15,000 vehicles/day and road tunnel wash water in Austria is not allowed to be discharged untreated (Meland, 2016). In Switzerland, if a new road project is projected to have >14,000 vehicles/day, runoff water will be required to discharge through an appropriate treatment system (Meland, 2016). Similarly, in Ireland, wash water from road tunnels in Ireland is sent to wastewater treatment plants (Meland, 2016). In Poland, permits are required for runoff and many require water treatment such as oil interceptors, small sedimentation tanks, infiltration ponds, wet ponds, and post-construction discharge monitoring for sediment (100 mg/L) and hydrocarbons (15 mg/L; Meland, 2016). In the US, California has proposed regulating microplastics (California State Water Resources Control Board, 2023) and has finalized a regulation that lists vehicle tires containing 6PPD under its Safer Consumer Products regulations (https://dtsc.ca.gov/scp/motor_vehicle_tires_containing_6ppd/). For particles associated with artificial turf and crumb rubber, there is recognition globally of the risks associated with the microplastics, PAHs, heavy metals, and PFAS chemicals of these rubber particles, though few countries and jurisdictions, with perhaps exception of the EU, have implemented adequate regulations and surveillance to address health risks and limit human exposure (Zuccaro et al., 2022).

## 8. Data gaps and research needs

While there has been an upsurge in tire related research, many data gaps remain that require attention to develop effective comprehensive approaches to managing risks (Wang et al., 2023). Research has been concentrated among US, China, and Europe with lesser focus in Africa or Asia (Wang et al., 2023). While our synthesis here is intended to be global, we acknowledge a limitation of our study in that there are fewer data and studies from developing countries and, as such, understanding the diverse nature of tire pollution and identifying effective solutions will require more work in regions outside the US, EU, and China where policies and infrastructure may not be adequate to address the complexities of risk management. Addressing the following data gaps and priority research areas will allow for more comprehensive and effective strategies to mitigate and prevent the effects of tire particles and tire-derived chemicals on the diverse terrestrial, aquatic, and marine ecosystems that become the sinks of these contaminants.

### 8.1. Tire production and use

Determining the complete and quantitative chemical composition of tire rubbers remains a critical and pressing research need, meriting systemic and intensive global effort. Study of how tire particles may vary across commonly used brands and tire types (*e.g*., snow tires, tires from hybrid and electric cars) and associated road wear, including particles from brake pads and mixtures with other particle types such as fibers being emitted from roadways (described in Brahney et al., 2021) is greatly needed. Data on which types of tires have the most use and tire wear particle emissions would help guide risk assessments of tire particles and tire-derived chemicals.

Given the ubiquitous nature of transportation, tires, and tire wear particles, this legacy pollutant footprint in the environment has the potential to be extensive. Developing multi-scalar and multi-disciplinary strategies for prioritizing mitigations for sensitive species population declines could support smarter transportation infrastructure planning.

### 8.2. Fate and transport

Given that there are thousands of compounds associated with tire leachate, including many compounds with considerable known toxicity, efforts to elucidate chemical structures of environmentally relevant tire contaminants remains a high priority. The fate and transport of tire wear particles is challenging due to special and temporal variation in the environment and by lack of standardized sampling methods as well as variability in tire composition and proprietary formulations which preclude adoption of analytical markers (Khan et al., 2024). Monitoring data of tire particles and many tire-derived chemicals remains limited, often due to a lack of reliable methods to extract and quantify them. Quantifying concentrations of tire particles and tire-related chemicals across environmental phases (*e.g*., urban runoff and wet and dry air deposition) are needed to understand the fate and transport of tire particles and tire-derived contaminants (Halle et al., 2020). Different sample matrices and the broad size distribution of tire microplastics require different methodologies to characterize and quantify tire microplastics in environmental samples, creating challenges for estimating their environmental fate and transport, as well as comparing data across studies (Luo et al., 2021; Wagner et al., 2018). Development of accurate sample collection, separation, and quantification methods for tire particles spanning the full particle size distribution and comprising different sample matrices (*e.g*., sediment, ambient surface water *vs*. effluent/stormwater, tissue, *etc*.) is needed to better characterize the fate and transport of tire particles (Baensch-Baltruschat et al., 2021; Halle et al., 2020; Luo et al., 2021).

Better characterization (*e.g*., size, shape, surface properties) of tire particles and determination of how particle characteristics affect particle fate and transport, as well as the formation and release of tire-related chemicals and their degradates is also needed. Larger tire particles are relatively easy to identify visually in environmental samples given their color and rubbery character (Gray et al., 2018; Miller et al., 2021), but are challenging to characterize analytically using commonly available instrumentation due to their high carbon content (Nava et al., 2021). Measuring the proportion of common environmental contaminants such as heavy metals and PAHs that tires are contributing to the environment relative to other sources would also help prioritize and focus source control efforts.

### 8.3. Biological and ecological effects

Like all microplastic types, it is critical to understand toxicological concerns centering on both the associated chemicals and the particles themselves. Toxicity testing is essential to fully understand both the physical and chemical impacts of tire particles. Toxicity experiments should reflect the size range of tire particles thought to be released into the environment, including the fine and ultrafine particles <1 μm. Research on the combined toxicity of tire-related chemicals and the particles themselves is also necessary to determine the true impact on both aquatic and terrestrial ecosystems, and humans. These studies should be coupled with investigations into the fate of tire-derived chemicals of high ecological concern, such as 6PPD and Zn, since it is not yet clear what chemical concentrations continue to be associated with particles once they are emitted into the environment and following weathering. As many tire-associated chemicals are not unique to tires and have well-understood hazards, researchers should draw upon existing toxicological data to help prioritize which chemical constituents found in tires may be most hazardous to biota. Studies of other micro- and nanoplastic particle types indicate that chemicals may largely leach out prior to internalization (Koelmans, 2015), yet because stabilizers such as 6PPD can be acutely toxic to sensitive species at low concentrations (Tian et al., 2021a), continued assessment of the fate and toxicity of chemicals used in the manufacture of tires and those suggested as replacements for toxic additives should be conducted. Additionally, the prediction of the toxic transformation products (*e.g*. 6PPD-quinone) will be challenging or impossible without additional manufacturer information which often is restricted or proprietary.

Determining differences in effects across different tire particle properties such as particle size, chemical composition, and level of degradation, as well as across both common lab species and more sensitive species will help inform risk assessment and prioritize remediation efforts. Species-specific ecotoxicology studies will help inform what corresponding habitats are most susceptible to tire emission exposure. Overall, the duration of most current toxicological investigations (usually only tens days) is one of the greatest drawbacks of these studies when the results need to be extrapolated to populations, communities, and ecosystems. Chronic, multigenerational exposures in the environment may lead to a reduction in the size of populations and to negative effects in communities and ecosystems. There is thus a need to conduct longer and *in-situ* exposures to discern community- and ecosystem-level effects of tire particles and associated chemicals over longer time horizons and across different ecosystems. New genomics tools may make these types of assessments more feasible and accurate (*e.g*. Giroux et al., 2023). Landscape-scale risk assessments would help focus source identification and reconnaissance studies. Few studies have addressed human health risks of tire wear particles or associated tire-derived chemicals (Wang et al., 2023).

### 8.4. Management and remediation

Toxic source control becomes more complex and challenging the further away from the source it travels. Focusing on prevention over remediation is therefore imperative. The range of solutions to plastic pollution, including tire particles, can be sorted by cost and harm, with design and material solutions having lowest cost and harm and recycling and cleanup at the high end of the cost-harm continuum (Erdle and Eriksen, 2023). Furthermore, because tires are a complex mixture of constituents, there must be tailored solutions for each category of pollutant and stage in the tire life cycle (Erdle and Eriksen, 2023). Understanding how watershed and climate characteristics, particularly under a rapidly changing climate, may drive tire emission pollution to vulnerable ecological areas will help determine where, when, and how often to mitigate tire wear emissions across various land uses.

The ability to measure and quantify tire emissions among water, sediment, air, and biota is critical to support the efficacy of toxic cleanup and reduction strategies. Emissions estimates based on measured data rather than summarized databases are critically needed to judge environmental loads locally and regionally (Khan et al., 2024). The variability in geographic attributes such as climate, geology, land use, vulnerable species distribution, population density, transportation infrastructure, and water quality regulations will determine the fate, transport, and biological exposure of tire emissions. For example, preliminary monitoring efforts have revealed that 6PPD-quinone is found ubiquitously in tires and on transportation pavement, yet its prevalence in receiving waters or sediments is more variable and less well understood (Rauert et al., 2022; Zeng et al., 2023).

Testing BMPs for tire particle and chemical removal efficiency to understand their stormwater treatment effectiveness is needed. Green infrastructure approaches like constructed wetlands, bioretention, and filtration features are promising BMPs for tire particle capture, but more research is needed on measuring efficacy based on multiple variables such as particle size and mobility as well as inflow rate and storm intensity (Wei et al., 2023). For BMPs such as bioretention gardens that are known to remove particles, further optimization (*e.g*., identifying optimal soil media mixtures and maintenance cycles; Bollman et al., 2019) is a critical need. Other emerging technologies have proved effective at removing other types of microplastics, including acoustic waves (Akiyama et al., 2020), electrocoagulation (Perren et al., 2018), photocatalysis (Koe et al., 2020), magnetic extraction (Grbic et al., 2019), and spider webs (Goßmann et al., 2022), and may also be promising for tire particles and/or tire-derived chemicals. It will also be necessary to identify the role of these BMPs as hot spots, biogeochemical reactors for transformations and/or entry pathways for tire particles or the pollutants they carry into local trophic chains.

### 8.5. Recycling, reuse, and disposal

Although the efforts to reuse and recycle tires at their end-of-life stage have increased (Shulman, 2019), new technologies and efforts are needed to increase the circularity of tire materials even further. Finding recycling, reuse, and disposal strategies for tires that minimize release of toxicants to the environment is critical. Thus, while more tire emissions will likely be generated in developed countries *versus* developing countries by virtue of the larger number of vehicles and more roads, the recycling infrastructure and/or pollution abatement services may be more readily available or effective. Conversely, developing countries may have fewer vehicles and therefore fewer emissions, the infrastructure and/or policy mechanisms may be lacking to deal with tire recycling or the associated pollution emissions. Technological solutions for capturing tire wear particles will likely progress more rapidly if the scientific community can effectively collaborate with the tire industry (Khan et al., 2024).

### 8.6. Data management and toxicity assessment

There are many needs to be addressed regarding data management associated with chemicals and toxicity associated with tires, whether they be initial constituents making up the tires or, as in the case of 6PPD-quinone, the transformation products associated with parent chemicals. For toxicological assessment, there is a critical need to elucidate both particle and leachate effects and to distinguish among the effects of single emerging chemicals as well as chemical cocktails and any transformation products (Khan et al., 2024). The aggregation of relevant data, whether experimental or predicted, has been addressed by systems including PubChem (https://pubchem.ncbi.nlm.nih.gov/), and ChemSpider (http://www.chemspider.com/; Pence and Williams, 2010) which provide access to 116 million and 128 million, respectively (as of December 2023). Currently, only the CompTox Chemicals Dashboard (Williams et al., 2017; https://comptox.epa.gov/dashboard/) provides access to an aggregated list of chemicals with associated, curated property and toxicity data for over 1.2 million chemical substances (as of December 2023). These data include physicochemical properties, fate and transport data, *in vivo* and *in vitro* toxicity, and exposure data. Chemicals can be searched individually or in batches of thousands of chemical identifiers (Lowe and Williams, 2021). The Dashboard can provide detailed information for chemicals such as 6PPD or summarized showing quantitative hazard data and *in vitro* bioactivity screening data. The Dashboard also lists tire crumb rubber chemicals based on data contained within the Federal Research Action Plan (FRAP) on Recycled Tire Crumb Used on Playing Fields and Playgrounds. Data can be harvested en masse by passing any list through the batch search. Note that many parameters are predicted *versus* measured which may represent significant uncertainty in ascertaining toxic effects. A new suite of prototype cheminformatics tools is publicly available with a hazard profile module allowing for comparison of toxicity effects across sets of chemicals including experimental and predicted human health effects, ecotoxicity, and fate (Vegosen and Martin, 2020).

## 9. Conclusion

Tires are a complex pollutant consisting of particles and chemicals that can be transported by air, water, and terrestrial pathways. The life cycle of tires spans production, use, and disposal. There are thousands of known and potentially toxic compounds contained in tires. Tires can pose risks to environmental and human health across the entire life cycle. Mitigating these risks is challenging given the volume of tires in use and produced as waste. Tire emissions may be in the form of whole tires, tire particles, and chemical compounds. Production and use of tires generates multiple heavy metals, radioactive compounds, and nutrients that can be toxic alone or as chemical cocktails. Tire particles emitted during use is a major component of microplastics in urban runoff and a source of unique and highly potent toxic substances, many of which are currently unknown or poorly described. Source control such as reducing the need for tires, recycling, or reuse is easier than remediating particles or chemicals that are released to the environment. Pollution from tires must be examined holistically across production, emissions, recycling, and disposal to develop effective management and remediation. There are many gaps in our knowledge about fate and transport, and toxicology of tire particles and leachates. Further research is needed to characterize the gamut of effects from tire pollution and to identify effective methods of remediation and risk management across environmental, socioeconomic, and political boundaries. However, it is currently clear that tire wear particles and their chemical cocktails are emerging contaminants of global concern, and that action should therefore be taken to reduce the risks to human health and the environment.

## Supplementary Material

SI

## Figures and Tables

**Table 1:** California waste tire use summary 2018*.

Use	Examples	Quantity (Metric Tons)
Combustion (export)	Burned at non-California facilities	130,000
Combustion	“Tire-derived fuel” burned at four California facilities	82,000
Landfill	Disposal, alternative daily cover	98,000
Reuse on vehicles	Used tires and retreads	82,000
Crumb/ground rubber	Rubberized asphalt pavement (60–67 %) Artificial turf infill (11–14 %) Mulch and ground covers (3–5 %) Molded & extruded products (19–20 %)	81,000
Civil engineering applications	Landfill structures, construction fill, vibration damping, and stormwater capture and treatment systems	4600
Other recycling	Unspecified	3100

Source: CalRecycle, 2019.

* Includes material imported from out of state. Reprinted from Moran et al. (2021).

**Table 2:** Fate of End of Life (Scrap) Tires in the United States and Europe in 2019. 76 % of scrap tires in the US are utilized in some fashion (not disposed of) and 95 % in the EU27 + NO+CH + RS + TR + UK.

Disposition (thousands of metric tons)	United States^1^	Europe^2^ EU27 + NO+CH + RS + TR + UK	Notes
Civil Engineering and Similar Uses	333.20 (8.2 %)	112.95 (3.2 %)	USA: includes Steel, Reclamation Projects, Agricultural, Baled Tires to Market and Punched Stamped. EU: includes public works and backfilling
Recycling	987.92 (24.4 %)	1841.20 (51.8 %)	USTMA does not use Recycling category. Ground Rubber included in total here. For EU, includes granulation and additions to cement manufacturing.
Energy Production	1493.98 (36.9 %)	1428.82 (40.2 %)	EU: Cement kilns 75 % and 25 % Urban Heating and power plants
Exported	125.19 (3.1 %)		
Other	119.64 (3.0 %)		
Land disposed	616.89 (15.2 %)		
Unknown/stocks	372.84 (9.2 %)	165.16 (4.6 %)	
Total	4049.67	3555.61	Totals are from Source Tables

Sources:

1 USTMA 2019. U.S. Scrap Tire Management Summary. U.S. Tire Manufacturer Association. Washington, D.C. 20005.

2 European Tyre and Rubber Manufacturing Association (ETRMA). Press Release: In Europe 95 % of all End of Life Tyres were collected and treated in 2019. https://www.etrma.org/news/in-europe-94-of-all-end-of-life-tyres-were-collected-and-treated-in-2019/

**Table 3:** Leaching of benzothiazole (Log KOW = 2.17, EpiSuite) from tire particles (TWP or crumb rubber) or tire road wear particles (TRWP).

Reference	Study design			Leachate conc., (μg/L)	Aqueous loading from tire (μg/g tire)		Leached into aqueous solution (% of tire concentration)	
	Particle generation; size	Leachate preparation	L:S* (L/kg) (conc., kg/L)	“Worn”	“Worn”	“New”	“Worn”	“New”
Unice et al. (2015)	TRWP generated in road simulator; no size information	Column, hard water, 30d, 20 °C	10 L/kg (0.1 kg/L)	1300^1^ TRWP	13 TRWP 26^4^ TWP	–	37^2^ TRWP	–
Halsband et al., 2020	Crumb rubber granulate; 1–2.8 mm	Batch, seawater, 14d, 20 °C	1000 L/kg (0.001 kg/L) highest L:S	68	68^1^	80^1^	62^1^	76^1^
			10 L/kg (0.1 kg/L)	1415	14^1^ (“pre-use”)	17^1^	13^1^ (“pre-use”)	16^1^
Halle et al., 2021	Tire tread on grindstone; avg. 176 um (worn), 210 um (“pristine”)	Batch, DI, 48 h, 25 °C	15 L/kg (0.067 kg/L)	254^1^	3.8	3.9	16	19
Capolupo et al., 2020	Tire derived granulate; 1–2 mm	Batch, freshwater (fw) or marine algal growth medium^3^, 14d, 25 °C	12.5 L/kg (0.08 kg/L)	2313 (fw) 1460 (marine)	29^1^ (fw) 18^1^ (marine)	–	–	–

1 Calculated from data provided in the study.

2 A new tire was used, but since particles were generated under more realistic conditions, TRWPs are classified here as “worn”.

3 Freshwater and marine algal growth media contained nutrients, trace metals and vitamins, which affected leaching of metals more than organic compounds (Capolupo et al., 2020).

4 Based on a 1:1 ratio of tread to road-derived minerals in TRWP (Peter et al., 2020).

* L:S = liquid-to-solid ratio.

**Table 4:** 6PPD-quinone aquatic toxicity in peer-reviewed literature. LC50 = median lethal concentration, C.L. = confidence limits.

Species	pH	Conductivity (μS/cm)	Temp. (°C)	Exp Time	Solution renewal	LC50 (μg/L; 95 % C.L.)	Ref.
*Oncorhynchus kisutch*	7.6–7.8	1250–1300	10–12	24 h	None	0.79 (0.6–1.0)^a^,*	1
*Oncorhynchus kisutch*	7.6–7.8	1250–1300	10–12	24 h	None	0.10 (0.08–0.11)^a,+^	2
*Danio rerio*	7.7 ± 0.0	3090 ± 200	25.9 ± 0.1	96 h	48 h	>70^b^,^^^	3
*Danio rerio*	7.4 ± 0.1	ISO^c^	27 ± 1	24 h	24 h	308.7 (258.3–368.9)^d^	4
*Oryzias latipes*	7.9 ± 0.1	3420 ±1100	24.4 ± 0.2	96 h	48 h	>40^b^,^^^	3
*Daphnia magna*	8.0–8.4	6380–6430	21.6–21.9	48 h	None	>60^b^,^^^	3
*Hyallela azteca*	8.0 ± 0.1	3100 ± 1100	23.5 ± 0.2	96 h	48 h	>90^b^,^^^	3
*Salvelinus fontinalis*	6.7 ± 0.1	131 ± 2.33^e^	10.3 ± 0.7	24 h	24 h	0.59 (0.48–0.63)^b^,^+^	5
*Oncorhynchus mykiss*	8.4 ± 0.5	132 ± 6.80^e^	12.8 ± 0.8	72 h	24 h	1.00 (0.95–1.05)^b^,^+^	5
*Oncorhynchus mykiss*	6.5 ± 0.0	143 ± 2	16 ± 1	96 h	48 h	2.26 (2.13–2.44)^a^	6
*Salvelinus alpinus*	8.4 ± 0.5	132 ± 6.80^e^	12.8 ± 0.8	96 h	24 h	>14.2^b^,^+^	5
*Acipenser transmontanus*	8.4 ± 0.5	132 ± 6.80^e^	12.8 ± 0.8	96 h	24 h	>12.7^b^,^+^	5
*Gobiocypris rarus*	8.0 ± 0.0	149 ± 2	25 ± 1	96 h	48 h	>500^a^	6
*Danio rerio*			28 ± 0.5	96 h	24 h	2200	7
*Oncorhynchus tshawytscha*	6.8–7.3	89.8 CaCO3	13.8 ± 0.3	24 h	none	67.3	8
*Oncorhynchus kisutch*	3.7–7.0	102 CaCO3	13.6 ± 0.3	24 h	none	0.041	8

1 Tian et al., 2021a;

2 Tian et al., 2022;

3 Hiki et al., 2021;

4 Varshney et al., 2022;

5 Brinkmann et al., 2022;

6 Di et al., 2022;

7 Peng et al., 2022;

8 Lo et al., 2023;

a Based on measured concentration at the start of exposure;

b Time weighted average concentration;

c ISO (International Standards Organization) Standard Fish Media;

d Based on nominal concentrations;

e Hardness as mg/L CaCO_3_;

* Concentrations measured using in-house standards without internal standard normalization. Tian et al. (2022) observed that use of a commercial standard yielded ~15-fold higher peak area response for the same 6PPD-quinone concentration in their in-house standard, and 6PPD-quinone recovery without internal standard normalization was ~60–70 %.;

+ Concentrations measured using a commercial standard;

^ Hiki et al. (2021) also created their own in-house standard and did not use an internal control when measuring exposure concentrations. Therefore, their reported concentrations may be overestimated, similar to Tian et al., 2021a.

**Table 5:** 6PPD-quinone occurrence in the environment.

Location and timing	Sample method	Sample type	Land use	Detected	Non-detected	6PPD-quinone (μg/L)	Coho NOEC^9^ 0.034 (μg/L)
Seattle, WA, US^1^ During storm event	Grab	Road surface	Urban highway	6	1	<0.05–1.27	Above
Seattle, WA, US^1^ During storm	Grab	Creek	Urbanized residential watersheds (Miller Creek, Longfellow Creek, Thornton Creek)			<0.02–0.21	Above
Seattle, WA, US^1^ Between storms	Grab	Creek	Urbanized residential watersheds (Miller Creek, Longfellow Creek, Thornton Creek)	0	15	NA	Below
Los Angeles, CA, US^1^ During storm	Grab	Road surface	Urban highway	2	0	0.49–0.74	Above
						
San Francisco Bay Area, CA, US^1^ During storm	Time Composite Samples	Creek	Urban and Reference sites	4	6	0.12–0.42	Above
Michigan, US^2^ Post storm 35 h	Grab	Creek	Various	2	17	0.012–0.037	Above
Michigan, US^2^ During storm	Grab	Road surface & puddles	Various	5	0	0.054–0.66	Above
Central California, US Dry Season	Grab	River		0	10	NA	NA
Central California, US Wet Season^3^	Grab	River		2	8	0.002–0.014	Below
Toronto, Canada^1^	Composite (42 h)	River	Urban, downstream of high-traffic corridor (Don River)			0.3–2.3	Above
Saskatoon, Canada^2^	Composite	Outfall	Urban (residential / light industrial)			0.086–1.40	Above
Saskatoon, Canada^5^	Composite (8–12 snow piles)	Snowmelt	Urban (residential / light industrial)			0.015–0.756	Above
Nanaimo, BC, Canada^3^	Grab	Creek	Not available	2	0	0.096–0.112	Above
Nanaimo, BC, Canada^6^	Grab	Stormwater	Not available	4		0.048–5.58	Above
Hong Kong^4^	Grab	Road surface	Dense traffic, urban area	9	0	0.021–0.243	Above
Brisbane, Australia^5^ post storm 18 days dry	Grab	Creek	Sub-urban (low density residential, open space)	9	0	0.0004–0.08	Above


1 Tian et al., 2022,

2 Nedrich, 2022;

3 Eurofin presentation to EPA group 2022;

4 Johannessen et al., 2021, 2022a, 2022b;

5 Challis et al., 2021;

6 Monaghan et al., 2021;

7 Cao et al., 2022;

8 Rauert et al., 2022;

9 Geometric mean of lowest observed effect concentration for mortality from Tian et al., 2022, Lo et al., 2022, Greer et al., 2023.

**Table 6:** Published toxicological studies on tire particles and leachates.

Reference	Tested species (* indicates those with observed significant effects)	Type of exposure			Authors consider exposures to be Environmentally Relevant Concentrations	Observed Affected Endpoints	Notes
		Micro particles	Nano particles	Leachates			
Carrasco-Navarro et al., 2022	California blackworm (*Lumbriculus variegatus*) Harlequin fly (*Chironomus riparius*)	x		x		None	
Garrard et al., 2022	*Scrobicularia plan *Ragworm *(Hediste diversicolor*)	x				Behavior Oxidative stress	
LaPlaca et al., 2022	*Mummichog *(Fundulus heteroclitus*)	x		x		DNA damage	Episodic exposures
Magni et al., 2022	*Zebrafish (Danio rerio) *Daphnia magna	x		x		Growth fecundity	Used end-of-life tires
Schell et al., 2022	*Daphnia magna Hyalella azteca Water louse (*Asellus aquaticus*) California blackworm (*Lumbriculus variegatus*)	x		x		Reproduction mortality	
Shin et al., 2022	*Rotifer *(Brachionus plicatilis*)			x		Mortality Oxidative stress	
Siddiqui et al., 2022	*Inland Silverside (*Menidia beryllina*) *Mysid shrimp (*Americamysis bahia*)	x	x	x	x	Behavior growth	
Carrasco-Navarro et al., 2021	*Harlequin fly (*Chironomus riparius*)	x			x	Gene expression	
Chibwe et al., 2022	*Fathead minnow (*Pimephales promelas*)			x		Development	
Cunningham et al., 2022	*Zebrafish (Danio rerio) *Daphnia magna	x	x	x		Behavior development mortality	Toxicity affected by changes in salinity
Halle et al., 2021	*Hyalella azteca	x		x		Growth mortality	
Koski et al., 2021	*Acartia tonsa Temora longicornis*	x			x	None	Used new tires, old tires, and rubber granules from artificial turfs
McIntyre et al., 2021	*Coho salmon (*Oncorhynchus kisutch*) Chum salmon (*Oncorhynchus keta*)			x	x	Mortality	
Selonen et al., 2021	Enchytraeid worm (*Enchytraeus crypticus*) *Springtail *(Folsomia candida*) Woodlouse (*Porcellio scaber*)	x		x		Reproduction mortality	
Sheng et al., 2021	*Earthworm *(Eisenia fetida*)	x				Oxidative stress	
Capolupo et al., 2020	*Mediterranean mussel (*Mytilus galloprovincialis*)			x		Reproduction	
Ding et al., 2020, 2023	*Soil worm *(Enchytraeus crypticus*)	x		x	x	Intestinal pathology, mortality	Tire brand-specific toxicity
Kolomijeca et al., 2020	*Fathead minnow (*Pimephales promelas*)			x		Reproduction Growth	Toxicity affected by temperature and mechanical stress
Khan et al., 2019	*Hyalella azteca	x				Mortality Growth Reproduction	
Pochron et al., 2018	*Earthworm *(Eisenia fetida*)	x				Mortality Growth	
Redondo-Hasselerharm et al., 2018	*Gammarus pulex Asellus aquaticus* Tubifex *spp. Lumbriculus variegatus*.	x				None	
Pochron et al., 2017	*Earthworm *(Eisenia fetida*)	x				Growth	
Panko et al., 2013	*Hyalella azteca Ceriodaphnia dubia* *Chironomus dilutus *Fathead minnow (*Pimephales promelas*)	x				Growth	Mimicked abrasion of tires to produce particles
Zhao et al., 2011	*Soil nematodes	x				Abundance Community structure	
Wik et al., 2009	*Ceriodaphnia dubia			x		Reproduction	

## Data Availability

No data was used for the research described in the article.

## HIGHLIGHTS

- Billions of tires are produced each year and hundreds of millions of tires become waste.
- Tires are a complex source of pollutants including whole tires, particles, compounds, and chemicals.
- As they wear, tires emit pollutants *via* atmospheric, aquatic, and terrestrial pathways.
- Tire wear pollutants represent an environmental and human health risk.
- Comprehensive clean-up solutions are needed to reduce the risk of tire wear pollutants.
