# Supplementary material for: Where the rubber meets the road: Emerging environmental impacts of tire wear particles and their chemical cocktails
Source: Sci Total Environ. Author manuscript; Available in PMC 2025 Jun 1. (PMC11214769; doi:10.1016/j.scitotenv.2024.171153)
Supplement: SI [file NIHMS1984980-supplement-SI.docx]

**Supplementary Table 1.** Chemical compounds found in tires and environmental samples. The aquatic toxicity scores were produced using a prototype computational tool developed by the USEPA (Williams et al., provisional version in security clearance mode). Toxicity is indicated as low (L), medium (M), high (H), very high (VH), and no data (ND). Bolded text indicates an authoritative result, plain text indicates screening results and italics indicate modeled results using computational prediction models. Reference column indicates the publication where the observed concentration was reported.

| **Compound** | **CAS # and (DTXSID)** | | **Industrial Use** | **Sample Type** | | **Observed Concentration** | | **Analytical Method** | | | **Field Sample Collection Region** | | **Aquatic Toxicity (acute/**  **chronic)** | | **Reference** |
| --- | --- | --- | --- | --- | --- | --- | --- | --- | --- | --- | --- | --- | --- | --- | --- |
| **Tire Chemical Additives** | | | | | | | | | | | | | | | |
| Acetophenone | 98-86-2  (DTXSID6021828) | |  | Field Crumb Rubber | | 0.22-540 mg/kg | | GC/MS/MS | | | Norway | | **L/**ND | | (Halsband et al., 2020) |
|  |  |  |  | Commercial Crumb Rubber | | -0.37 mg/kg | | GC/MS/MS | | | NA | |  |  | (Halsband et al., 2020) |
| N,N-Dicyclohexylmethylamine (DCA) | 7560-83-0  (DTXSID6044727) | |  | Snowmelt | | 3.2 – 10 ng/L | | UHPLC-QMS | | | Canada | | H/*H* | | (Challis et al., 2021) |
|  |  |  |  | Stormwater | | 8 – 60 ng/L | | UHPLC-QMS | | | Canada | |  |  | (Challis et al., 2021) |
| N,N’-Dicyclohexylurea (DCU) | 2387-23-7  (DTXSID3062366) | |  | Snowmelt | | <1.2 – 130 ng/L | | UHPLC-QMS | | | Canada | | *M/H* | | (Challis et al., 2021) |
|  |  |  |  | Stormwater | | 148 – 1890 ng/L | | UHPLC-QMS | | | Canada | |  |  | (Challis et al., 2021) |
|  |  |  |  | Surface water | | 0.9-1.8 ng/L | | HPLC-TOF-MS | | | NA | |  |  | (Tian et al., 2020) |
|  |  |  |  | Surface water (during storm events) | | <8 – 253 ng/L | | HPLC-TQMS | | | Australia | |  |  | (Rauert et al., 2022) |
| hexa(methoxymethyl)melamine (HMMM) | 3089-11-0  (DTXSID9027520) | | Crosslinking agent    tire bonding agent | Stormwater | | 0.5 – 130 ng/L | | reversed-phase HPLC-MS/MS | | | USA | | L/*M* | | (Hou et al., 2019) |
|  |  |  |  | Surface water (during storm events) | | <0.5 – 248 ng/L | | HPLC-TQMS | | | Australia | |  |  | (Rauert et al., 2022) |
|  |  |  |  | Road runoff | | 2,000-6,500 ng/L | | HPLC-TOF-MS | | | NA | |  |  | (Peter et al., 2018) |
|  |  |  |  | TWP leachates | | 4,000-8,800 ng/L | | HPLC-TOF-MS | | | NA | |  |  | (Peter et al., 2018) |
|  |  |  |  | Surface water | | 10-54 ng/L | | HPLC-TOF-MS | | | NA | |  |  | (Peter et al., 2018) |
|  |  |  |  | Surface water | | 1.0-28 ng/L | | HPLC-TOF-MS | | | NA | |  |  | (Tian et al., 2020) |
|  |  |  |  | Surface water | | 0.06-0.53 ug/L | | LC-PDA-QTOF-MS | | | Germany | |  |  | (Alhelou et al., 2019) |
|  |  |  |  | Surface water | | 1.05 – 2.26 μg/L | | UHPLC-HRMS | | | Canada | |  |  | (Johannessen et al., 2021) |
| (Diformylated HMMM) | 38222-55-8 (DTXSID001017828) | |  | Surface water | | <LOD-0.03 ug/L | | UHPLC-HRMS | | | Canada | |  | | (Johannessen et al., 2021) |
| D501 (C_14_H_26_N_6_O_6_) | NA  (DTXSID501352912) | |  | Surface water | | <LOD-1.42 ug/L | | UHPLC-HRMS | | | Canada | |  | | (Johannessen et al., 2021) |
| Penta(Methoxymethyl)Melamine | 3169-30-0  (DTXSID30596249) | |  | Surface water | | 0.07-7.09 ug/L | | UHPLC-HRMS | | | Canada | | *H/*ND | | (Johannessen et al., 2021) |
|  |  |  |  | Surface water | | 0-0.07 ug/L | | LC-PDA-QTOF-MS | | | Germany | |  |  | (Alhelou et al., 2019) |
| D420 (C_13_H_26_N_6_O_6_) | 251294-81-2  (DTXSID501353172) | |  | Surface water | | <LOD-0.11 ug/L | | UHPLC-HRMS | | | Canada | |  | | (Johannessen et al., 2021) |
| D411  (C_13_H_24_N_6_O_6_) | NA  (DTXSID901352914) | |  | Surface water | | <LOD-1.01 ug/L | | UHPLC-HRMS | | | Canada | |  | | (Johannessen et al., 2021) |
| D401  (C_12_H_22_N_6_O_5_) | 2415923-12-3  (DTXSID601352915) | |  | Surface water | | <LOD-4.01 ug/L | | UHPLC-HRMS | | | Canada | |  | | (Johannessen et al., 2021) |
|  |  |  |  | Surface water | | 0.42-3.65 ug/L | | LC-PDA-QTOF-MS | | | Germany | |  |  | (Alhelou et al., 2019) |
| Tetra(Methoxymethyl)Melamine | 50322-79-7  (DTXSID30598925) | |  | Surface water | | <LOD-0.66 ug/L | | UHPLC-HRMS | | | Canada | | *H/*ND | | (Johannessen et al., 2021) |
|  |  |  |  | Surface water | | 0.08-0.25 ug/L | | LC-PDA-QTOF-MS | | | Germany | |  |  | (Alhelou et al., 2019) |
| D330  (C_12_H_24_N_6_O_6_) | NA  (DTXSID301352916) | |  | Surface water | | 0.43-0.81 ug/L | | UHPLC-HRMS | | | Canada | |  | | (Johannessen et al., 2021) |
| D311  (C_11_H_20_N_6_O_5_) | NA  (DTXSID001352917) | |  | Surface water | | <LOD-1.55 ug/L | | UHPLC-HRMS | | | Canada | |  | | (Johannessen et al., 2021) |
| D310  (C_10_H_20_N_6_O_4_) | NA  (DTXSID701352918) | |  | Surface water | | 0.37-8.06 ug/L | | UHPLC-HRMS | | | Canada | |  | | (Johannessen et al., 2021) |
| Tri(Methoxymethyl)Melamine | 2420-27-1  (DTXSID70947014) | |  | Surface water | | 0.11-2.10 ug/L | | UHPLC-HRMS | | | Canada | | *M/H* | | (Johannessen et al., 2021) |
| D210  (C_8_H_16_N_6_O_3_) | NA  (DTXSID401352919) | |  | Surface water | | 0.30-1.43 ug/L | | UHPLC-HRMS | | | Canada | |  | | (Johannessen et al., 2021) |
|  |  |  |  | Surface water | | 0.02-0.24 ug/L | | LC-PDA-QTOF-MS | | | Germany | |  |  | (Alhelou et al., 2019) |
| Di(Methoxymethyl)Melamine | 2415923-14-5  (DTXSID701352920) | |  | Surface water | | 0.69-11.16 ug/L | | UHPLC-HRMS | | | Canada | |  | | (Johannessen et al., 2021) |
|  |  |  |  | Surface water | | 0.11-0.76 ug/L | | LC-PDA-QTOF-MS | | | Germany | |  |  | (Alhelou et al., 2019) |
| Mono(Methoxymethyl)Melamine | 4261-70-5  (DTXSID20962611) | |  | Surface water | | <LOQ-0.78 ug/L | | UHPLC-HRMS | | | Canada | | *L/H* | | (Johannessen et al., 2021) |
|  |  |  |  | Surface water | | 0.11-0.75 ug/L | | LC-PDA-QTOF-MS | | | Germany | |  |  | (Alhelou et al., 2019) |
| Melamine | 108-78-1  (108-78-1) | |  | Surface water | | 0.40-19.90 ug/L | | LC-PDA-QTOF-MS | | | Germany | | L/L | | (Alhelou et al., 2019) |
| 1-Cyclohexyl-3-phenylurea (CPU) | 886-59-9  (DTXSID90877268) | |  | Snowmelt | | 4.8 – 125 ng/L | | UHPLC-QMS | | | Canada | | M/ND | | (Challis et al., 2021) |
|  |  |  |  | Stormwater | | 59 – 198 ng/L | | UHPLC-QMS | | | Canada | |  |  | (Challis et al., 2021) |
|  |  |  |  | Surface water (during storm events) | | <0.01 – 239 ng/L | | HPLC-TQMS | | | Australia | |  |  | (Rauert et al., 2022) |
| *N*-cyclohexyl-1,3-benzothiazole-2-amine (NCBA)] | 28291-75-0  (DTXSID50891506) | |  | Stormwater | | ND | | reversed-phase  HPLC-MS/MS | | | USA | | *L/*ND | | (Hou et al., 2019) |
|  |  |  |  | Receiving water during storm | | <0.4 – 10 ng/L | | HPLC-TQMS | | | Australia | |  |  | (Rauert et al., 2022) |
|  |  |  |  | Aquatic sediments | | 2.3-17 ng/g | | GC-FPD | | | Japan | |  |  | (Kumata et al., 2002) |
| 1-octanethiol | 111-88-6  (DTXSID4026894) | |  | Tire wear particles | | 1.82 μg/g | | GC-MS | | | NA | | VH/VH | | (Halle et al., 2021) |
|  |  |  |  | Pristine tire particles | | 14.96 μg/g | | GC-MS | | |  |  |  |  | (Halle et al., 2021) |
| 1-indanone | 83-33-0  (DTXSID1058892) | |  | Tire wear particles | | 6.46 μg/g | | GC-MS | | | NA | | *M/*ND | | (Halle et al., 2021) |
|  |  |  |  | Pristine tire particles | | 5.26 μg/g | | GC-MS | | |  |  |  |  | (Halle et al., 2021) |
| n-cyclohexylformamide | 766-93-8  (DTXSID40227416) | | additive | Field Crumb Rubber | | 2-9 mg/kg | | GC/MS/MS | | | Norway | | *M*/ND | | (Halsband et al., 2020) |
|  |  |  |  | Commercial Crumb Rubber | | 22 mg/kg | | GC/MS/MS | | | NA | |  |  | (Halsband et al., 2020) |
| 6PPD-quinone | 2754428-18-5  (DTXSID301034849) | | NA | Surface water | | 210 – 760 ng/L | | UPLC-HRMS | | | Canada | | *H/*ND | | (Johannessen et al., 2022a) |
|  |  |  |  | Roadway Runoff | | 0.21–2.43 μg/L | | UHPLC-TQMS | | | Hong Kong | |  |  | (Cao et al., 2022) |
|  |  |  |  | Stormwater Runoff | | 86–1400 ng/L | | UHPLC-TQMS | | | Canada | |  |  | (Challis et al., 2021) |
|  |  |  |  | Receiving water during storm | | 0 .38 – 88 ng/L | | HPLC-TQMS | | | Australia | |  |  | (Rauert et al., 2022) |
|  |  |  |  | Surface water | | 0.05 – 0.72 μg/L | | UHPLC-HRMS | | | Canada | |  |  | (Johannessen et al., 2021) |
|  |  |  |  | Roadside soil | | 9.50–936 ng/g | | UHPLC-TQMS | | | Hong Kong | |  |  | (Cao et al., 2022) |
|  |  |  |  | Road dust | | 116 – 1238 ng/g | | HPLC-MS/MS | | | Tokyo | |  |  | (Hiki and Yamamoto, 2022) |
|  |  |  |  | Air | | 0.54–13.8 pg/m3 | | UHPLC-TQMS | | | Hong Kong | |  |  | (Cao et al., 2022) |
|  |  |  |  | Road dust | | 10.5 – 509 ng/g | | HPLC-MS/MS | | | China | |  |  | (Deng et al., 2022) |
|  |  |  |  | WWTP | | <LOQ-0.105 μg/L | | UPLC-TOF-MS | | | Germany | |  |  | (Seiwert et al., 2022) |
|  |  |  |  | PM_2.5_ | | 2.44-7,250 pg/m3 | | HPLC-Orbitrap-MS | | | China | |  |  | (Wang et al., 2022) |
|  |  |  |  | Snowmelt | | 17 – 536 ng/L | | UHPLC-QMS | | | Canada | |  |  | (Challis et al., 2021) |
| 2-(isopropylamino)-5-(phenylamino)cyclohexa-2,5-diene1,4-dione (IPPD-Q) | NA  (DTXSID401352921) | | NA | PM_2.5_ | | <LOQ-3,250 pg/m3 | | HPLC-Orbitrap-MS | | | China | | ND/ND | | (Wang et al., 2022) |
| 2-(cyclohexylamino)-5-(phenylamino)cyclohexa-2,5-diene1,4-dione (CPPD-Q) | NA  (DTXSID101352922) | | NA | PM_2.5_ | | <LOQ-1,380 pg/m3 | | HPLC-Orbitrap-MS | | | China | | ND/ND | | (Wang et al., 2022) |
| 2,5-bis((5-methylhexan-2-yl)amino)cyclohexa-2,5-diene-1,4dione (77PD-Q) | NA  (DTXSID801352923) | | NA | PM_2.5_ | | <LOQ-2,990 pg/m3 | | HPLC-Orbitrap-MS | | | China | | ND/ND | | (Wang et al., 2022) |
| 2,5-bis(phenylamino)cyclohexa-2,5-diene-1,4-dione (DPPD-Q) | 3421-08-7  (DTXSID10279149) | | NA | PM_2.5_ | | <LOQ-2,100 pg/m3 | | HPLC-Orbitrap-MS | | | China | | ND/ND | | (Wang et al., 2022) |
| 2,5-bis(o-tolylamino)cyclohexa-2,5-diene-1,4-dione (DTPD-Q) | NA  (DTXSID501352924) | | NA | PM_2.5_ | | <LOQ-3.23 pg/m3 | | HPLC-Orbitrap-MS | | | China | | ND/ND | | (Wang et al., 2022) |
| 4-hydroxydiphenylamine (4-HDPA) | 122-37-2  (DTXSID5037739) | | NA | WWTP | | <LOQ-0.070 μg/L | | UPLC-TOF-MS | | | Germany | | *H*/ND | | (Seiwert et al., 2022) |
|  |  |  |  | Cured tire tread material | | <5 ug/g | | LC/MS/MS | | | NA | |  |  | (Unice et al., 2015) |
|  |  |  |  | Tire road wear particles leachate | | <0.1-42 ug/g | | LC/MS/MS | | | NA | |  |  | (Unice et al., 2015) |
|  |  |  |  | Tire road wear particles sediment incubator test | | 0.5 - <11 ug/g | | LC/MS/MS | | | NA | |  |  | (Unice et al., 2015) |
| 4-Aminodiphenylamine | 101-54-2  (DTXSID7025895) | |  | Cured tire tread material | | 26 ug/g | | LC/MS/MS | | | NA | | VH/VH | | (Unice et al., 2015) |
|  |  |  |  | Tire road wear particles leachate | | <0.03-17 ug/g | | LC/MS/MS | | | NA | |  |  | (Unice et al., 2015) |
|  |  |  |  | Tire road wear particles sediment incubator test | | <2 - <11 ug/g | | LC/MS/MS | | | NA | |  |  | (Unice et al., 2015) |
| 4-Nitrodiphenylamine | 836-30-6  (DTXSID8027323) | |  | Tire road wear particles leachate | | <0.1 ug/g | | LC/MS/MS | | | NA | | VH/VH | | (Unice et al., 2015) |
|  |  |  |  | Tire road wear particles sediment incubator test | | <6 - < ug/g | | LC/MS/MS | | | NA | |  |  | (Unice et al., 2015) |
| Diphenylamine | 122-39-4  (DTXSID4021975) | |  | Cured tire tread material | | 1.0 ug/g | | LC/MS/MS | | | NA | | **VH/VH** | | (Unice et al., 2015) |
|  |  |  |  | Tire road wear particles leachate | | <0.01-2 ug/g | | LC/MS/MS | | | NA | |  |  | (Unice et al., 2015) |
|  |  |  |  | Tire road wear particles sediment incubator test | | <0.2 - <1 ug/g | | LC/MS/MS | | | NA | |  |  | (Unice et al., 2015) |
| **Antioxidants and vulcanization agents** | | | | | | | | | | | | | | | |
| *N*-1,3-dimethylbutyl-*N*′-phenyl-*p-*phenylenediamine nitrosamine (6PPD) | 793-24-8  (DTXSID9025114) | | Antiozonant Antioxidant | | Air | | 0.82–6.30 pg/m3 | | UHPLC-TQMS | Hong Kong | | VH/VH | | (Cao et al., 2022) | |
|  |  |  |  |  | Roadside Soil | | 31.4–831 ng/g | | UHPLC-TQMS | Hong Kong | |  |  | (Cao et al., 2022) | |
|  |  |  |  |  | Road dust | | 45 – 1068 ng/g | | HPLC-MS/MS | Tokyo | |  |  | (Hiki and Yamamoto, 2022) | |
|  |  |  |  |  | Roadway Runoff | | 0.21–2.71 μg/L | | UHPLC-TQMS | Hong Kong | |  |  | (Cao et al., 2022) | |
|  |  |  |  |  | Road dust | | 15.1 – 1508 ng/g | | HPLC-MS/MS | China | |  |  | (Deng et al., 2022) | |
|  |  |  |  |  | PM_2.5_ | | 1.02-9,340 pg/m3 | | HPLC-Orbitrap-MS | China | |  | | (Wang et al., 2022) | |
|  |  | |  | | Uncured tire tread material | | 2200 ug/g | | LC/MS/MS | NA | |  | | (Unice et al., 2015) | |
|  |  | |  | | Cured tire tread material | | 1200 ug/g | | LC/MS/MS | NA | |  | | (Unice et al., 2015) | |
|  |  | |  | | Tire road wear particles leachate | | 0.013-1000 ug/g | | LC/MS/MS | NA | |  | | (Unice et al., 2015) | |
|  |  | |  | | Tire road wear particles sediment incubator test | | 0.5-120 ug/g | | LC/MS/MS | NA | |  | | (Unice et al., 2015) | |
| N-isopropyl-N′-phenyl-1,4-phenylenediamine (IPPD) | 101-72-4  (DTXSID1025485) | | Antiozonant Antioxidant | | Road dust | | <LOQ – 247 ng/g | | HPLC-MS/MS | China | | **VH/VH** | | (Deng et al., 2022) | |
|  |  |  |  |  | PM_2.5_ | | 0.49-3,690 pg/m3 | | HPLC-Orbitrap-MS | China | |  |  | (Wang et al., 2022) | |
| N, N′-diphenyl-p-phenylenediamine (DPPD) | 74-31-7  (DTXSID9020538) | | Antiozonant Antioxidant | | Road dust | | <LOQ – 153 ng/g | | HPLC-MS/MS | China | | L**/M** | | (Deng et al., 2022) | |
|  |  |  |  |  | PM_2.5_ | | 0.69-2,590 pg/m3 | | HPLC-Orbitrap-MS | China | |  |  | (Wang et al., 2022) | |
| N-phenyl-N’-cyclohexyl-p-phenylenediamine (CPPD) | 101-87-1  (DTXSID2051508) | | Antiozonant Antioxidant | | PM_2.5_ | | <LOQ-672 pg/m3 | | HPLC-Orbitrap-MS | China | | L**/***VH* | | (Wang et al., 2022) | |
| (1,4-dimethylpentyl)-N'-phenylbenzene-1,4-diamine (7PPD) | 3081-01-4  (DTXSID5027516) | | Antiozonant Antioxidant | | PM_2.5_ | | <LOQ-75.0 pg/m3 | | HPLC-Orbitrap-MS | China | | ND/ND | | (Wang et al., 2022) | |
| N,N'-Bis(1,4-dimethylpentyl)-P-phenylenediamine (77PD) | 3081-14-9  (DTXSID2024618) | | Antiozonant Antioxidant | | PM_2.5_ | | <LOQ-4,150 pg/m3 | | HPLC-Orbitrap-MS | China | | VH/VH | | (Wang et al., 2022) | |
| N,N''-Bis(methylphenyl)-1,4-benzenediamine (DTPD) | 15017-02-4  (DTXSID1065843) | | Antiozonant Antioxidant | | PM_2.5_ | | <LOQ-27.1 pg/m3 | | HPLC-Orbitrap-MS | China | | *VH/VH* | | (Wang et al., 2022) | |
| N,N'-Di-b-naphthyl-p-phenylenediamine (DNPD) | 93-46-9  (DTXSID3020918) | | Antiozonant Antioxidant | | PM_2.5_ | | <LOQ-61.3 pg/m3 | | HPLC-Orbitrap-MS | China | | H/*VH* | | (Wang et al., 2022) | |
| 1,3-diphenylguanidine (DPG) | 102-06-7  (DTXSID3025178) | | vulcanization accelerator | | Stormwater | | 5 – 540 ng/L | | reversed-phase  HPLC-MS/MS | USA | | H/**H** | | (Hou et al., 2019) | |
|  |  |  |  |  | Snowmelt | | 452 – 1607 ng/L | | UHPLC-QMS | Canada | |  |  | (Challis et al., 2021) | |
|  |  |  |  |  | Surface water (during storm events) | | 13 – 1079 ng/L | | HPLC-TQMS | Australia | |  |  | (Rauert et al., 2022) | |
|  |  |  |  |  | Road runoff | | ~1800 ng/L | | HPLC-TOF-MS | NA | |  |  | (Peter et al., 2018) | |
|  |  |  |  |  | Surface water | | ~20 ng/L | | HPLC-TOF-MS | NA | |  |  | (Peter et al., 2018) | |
|  |  |  |  |  | Surface water | | 0.9-93 ng/L | | HPLC-TOF-MS | NA | |  |  | (Tian et al., 2020) | |
|  |  |  |  |  | Uncured tire tread material | | 1400 ug/g | | LC/MS/MS | NA | |  |  | (Unice et al., 2015) | |
|  |  |  |  |  | Cured tire tread material | | 650 ug/g | | LC/MS/MS | NA | |  |  | (Unice et al., 2015) | |
|  |  |  |  |  | Tire road wear particles leachate | | 8.1-310 ug/g | | LC/MS/MS | NA | |  |  | (Unice et al., 2015) | |
|  |  |  |  |  | Tire road wear particles sediment incubator test | | 3.0-98 ug/g | | LC/MS/MS | NA | |  |  | (Unice et al., 2015) | |
|  |  |  |  |  | Surface water | | 0.05 – 0.74 μg/L | | UHPLC-QMS | Canada | |  |  | (Johannessen et al., 2021) | |
| Aniline | 62-53-3  (DTXSID8020090) | |  | | Cured tire tread material | | 11 ug/g | | LC/MS/MS | NA | |  | | (Unice et al., 2015) | |
|  |  |  |  |  | Tire road wear particles leachate | | <1-92 ug/g | | LC/MS/MS | NA | |  |  | (Unice et al., 2015) | |
|  |  |  |  |  | Tire road wear particles sediment incubator test | | 5.8-48 ug/g | | LC/MS/MS | NA | |  |  | (Unice et al., 2015) | |
|  |  |  |  |  | Tire crumb rubber | | 0.67-3.8 mg/kg | | GC/MS/MS | NA | |  |  | (US EPA, 2019) | |
| Benzothiazole | 95-16-9  (DTXSID7024586) | | Rubber accelerator | | Freshwater tire particle leachate | | 2,313 μg/L | | GC-MS | NA | | **M/**M | | (Capolupo et al., 2020) | |
|  |  |  |  |  | Marine tire particle leachate | | 1,460 μg/L | |  |  |  |  |  |  |  |
|  |  |  |  |  | Tire wear particle leachate | | 346 – 19,432 μg/g | | HPLC–MS/MS |  |  |  |  | (Jeong et al., 2022) | |
|  |  |  |  |  | Tire wear particles | | 23.33 μg/g | | GC-MS | NA | |  |  | (Halle et al., 2021) | |
|  |  |  |  |  | Pristine tire particles | | 20.22 μg/g | |  |  |  |  |  |  |  |
|  |  |  |  |  | Field Crumb Rubber | | 37-110 mg/kg | | GC/MS/MS | Norway | |  |  | (Halsband et al., 2020) | |
|  |  |  |  |  | Field Crumb Rubber Seawater Leachate | | 27-1415 µg/L | |  |  |  |  |  |  |  |
|  |  |  |  |  | Commercial Crumb Rubber | | 105 mg/kg | | GC/MS/MS | NA | |  |  | (Halsband et al., 2020) | |
|  |  |  |  |  | Commercial Crumb Rubber Seawater Leachate | | 80-693 µg/L | |  |  |  |  |  |  |  |
|  |  |  |  |  | Cured tire tread material | | <10 ug/g | | LC/MS/MS | NA | |  |  | (Unice et al., 2015) | |
|  |  |  |  |  | Tire road wear particles leachate | | <0.5-35 ug/g | | LC/MS/MS | NA | |  |  | (Unice et al., 2015) | |
|  |  |  |  |  | Tire road wear particles sediment incubator test | | 11 - <160 ug/g | | LC/MS/MS | NA | |  |  | (Unice et al., 2015) | |
|  |  |  |  |  | Road Dust | | <LOQ – 8,599 ng/g | | HPLC-MS/MS | China | |  |  | (Deng et al., 2022) | |
|  |  | |  | | Tire crumb rubber | | 11-79 mg/kg | | GC/MS/MS | NA | |  | | (US EPA, 2019) | |
| 2-amino-benzothiazole | 136-95-8  (DTXSID1024467) | |  | | Stormwater | | 0.5 – 11 ng/L | | reversed-phase  HPLC-MS/MS | USA | | **M**/**M** | | (Hou et al., 2019) | |
| 2-hydroxy-benzothiazole | 934-34-9  (DTXSID6061315) | |  | | Stormwater | | 18 – 160 ng/L | | reversed-phase  HPLC-MS/MS | USA | | L/ND | | (Hou et al., 2019) | |
|  |  |  |  |  | Road Dust | | <LOQ – 4,026 ng/g | | HPLC-MS/MS | China | |  |  | (Deng et al., 2022) | |
| 2-benzothiazolone | 934-34-9  (DTXSID6061315) | |  | | Cured tire tread material | | 5.2 ug/g | | LC/MS/MS | NA | | ND/ND | | (Unice et al., 2015) | |
|  |  |  |  |  | Tire road wear particles leachate | | 0-20 ug/g | | LC/MS/MS | NA | |  |  | (Unice et al., 2015) | |
| 2-Benzothiazolesulfonic acid | 941-57-1  (DTXSID80240528) | |  | | Tire road wear particles leachate | | 31-55 ug/g | | LC/MS/MS | NA | | ND/ND | | (Unice et al., 2015) | |
|  |  |  |  |  | Tire road wear particles sediment incubator test | | 1.1-27 ug/g | | LC/MS/MS | NA | |  |  | (Unice et al., 2015) | |
| Cyclohexylamine | 108-91-8  (DTXSID1023996) | |  | | Cured tire tread material | | 69 ug/g | | LC/MS/MS | NA | | **M/**L | | (Unice et al., 2015) | |
|  |  |  |  |  | Tire road wear particles leachate | | <4-33 ug/g | | LC/MS/MS | NA | |  |  | (Unice et al., 2015) | |
|  |  |  |  |  | Tire road wear particles sediment incubator test | | 7.0-78 ug/g | | LC/MS/MS | NA | |  |  | (Unice et al., 2015) | |
| 2-mercaptobenzothiazole | 149-30-4  (DTXSID1020807) | | vulcanizer | | Field crumb rubber | | 1–205 μg/g | | GC-MS/MS | Portugal | | **VH**/**VH** | | (Celeiro et al., 2021) | |
|  |  |  |  |  | Surface water | | 3.0-170 ng/L | | HPLC-TOF-MS | NA | |  |  | (Tian et al., 2020) | |
|  |  |  |  |  | Cured tire tread material | | 9.4 ug/g | | LC/MS/MS | NA | |  |  | (Unice et al., 2015) | |
|  |  |  |  |  | Tire road wear particles leachate | | <0.1 – 7.5 ug/g | | LC/MS/MS | NA | |  |  | (Unice et al., 2015) | |
|  |  |  |  |  | Tire road wear particles sediment incubator test | | <6 - <27 ug/g | | LC/MS/MS | NA | |  |  | (Unice et al., 2015) | |
| 2-(4-morpholinyl)benzothiazole | 4225-26-7  (DTXSID90891505) | |  | | Stormwater | | 1.5 – 24 ng/L | | reversed-phase  HPLC-MS/MS | USA | | *M/*ND | | (Hou et al., 2019) | |
|  |  |  |  |  | Street dust | | 27 ng/g | | GC-FPD | Japan | |  |  | (Kumata et al., 1996) | |
|  |  |  |  |  | River sediment | | 2.5-5.1 ng/g | | GC-FPD | Japan | |  |  | (Kumata et al., 1996) | |
|  |  |  |  |  | Particles in surface water | | 0-29 ng/L | | GC-FPD | Japan | |  |  | (Kumata et al., 1996) | |
|  |  |  |  |  | Atmospheric aerosol | | 5.9 pg/m^3^ | | GC-FPD | Japan | |  |  | (Kumata et al., 1996) | |
| 2-chlorobenzothiazole | 615-20-3  (DTXSID2052289) | |  | | Road Dust | | 298 – 12,798 ng/g | | HPLC-MS/MS | China | | H/*H* | | (Deng et al., 2022) | |
| 2-methylthio-benzothiazole | 615-22-5  (DTXSID70274236) | |  | | Road Dust | | <LOQ – 3,317 ng/g | | HPLC-MS/MS | China | | ND/ND | | (Deng et al., 2022) | |
|  |  |  |  |  | Cured tire tread material | | <3 ug/g | | LC/MS/MS | NA | |  |  | (Unice et al., 2015) | |
|  |  |  |  |  | Tire road wear particles leachate | | <0.1 - <4 ug/g | | LC/MS/MS | NA | |  |  | (Unice et al., 2015) | |
|  |  |  |  |  | Tire road wear particles sediment incubator test | | <6 - <27 ug/g | | LC/MS/MS | NA | |  |  | (Unice et al., 2015) | |
| 2,2′-Dithiobis(benzothiazole) | 120-78-5  (DTXSID1020146) | |  | | Cured tire tread material | | 2.1 ug/g | | LC/MS/MS | NA | | **VH/VH** | | (Unice et al., 2015) | |
|  |  |  |  |  | Tire road wear particles leachate | | <1 ug/g | | LC/MS/MS | NA | |  |  | (Unice et al., 2015) | |
| 2-morpholinothio-benzothiazole | 102-77-2  (DTXSID0021096) | |  | | Road Dust | | <LOQ – 29.6 ng/g | | HPLC-MS/MS | China | | VH**/H** | | (Deng et al., 2022) | |
| Benzothiazole-2-sulfonic acid | 941-57-1  (DTXSID80240528) | | Corrosion inhibitor | | Surface water | | 47-820 ng/L | | HPLC-TOF-MS | NA | | ND/ND | | (Tian et al., 2020) | |
| Benzotriazole | 95-14-7  (DTXSID6020147) | | Corrosion inhibitor | | Stormwater | | 3 – 88 ng/L | | reversed-phase  HPLC-MS/MS | USA | | **M/L** | | (Hou et al., 2019) | |
| Benzothiazole-2-sulfonic acid | 941-57-1  (DTXSID80240528) | | Corrosion inhibitor | | Surface water | | 2.2-6.3 ng/L | | HPLC-TOF-MS | NA | | ND/ND | | (Tian et al., 2020) | |
| 5-methyl-1*H*-benzotriazole | 136-85-6  (DTXSID1038743) | |  | | Stormwater | | 0.5 – 74 ng/L | | reversed-phase  HPLC-MS/MS | USA | | **M**/**M** | | (Hou et al., 2019) | |
|  |  |  |  |  | Surface water | | 0.4-27 ng/L | | HPLC-TOF-MS | NA | |  |  | (Tian et al., 2020) | |
| Butylhydroxytoluene | 128-37-0  (DTXSID2020216) | | antioxidant | | Field crumb rubber | | 0.1–2.0 μg/g | | GC-MS/MS | Portugal | | VH/VH | | (Celeiro et al. 2021) | |
| 4-tert-butyl phenol | 98-54-4  (DTXSID1020221) | | antioxidant | | Field crumb rubber | | 0.02–3.73 μg/g | | GC-MS/MS | Portugal | | **H**/M | | (Celeiro et al. 2021) | |
|  |  |  |  |  | Tire crumb rubber | | 9.8-30 mg/kg | | GC/MS/MS | NA | |  |  | (US EPA, 2019) | |
| N-cyclohexyl-1,3-benzothiazol-2-amine | 28291-75-0  (DTXSID50891506) | |  | | Tire road wear particles leachate | | <0.002-2 ug/g | | LC/MS/MS | NA | | ND/ND | | (Unice et al., 2015) | |
|  |  |  |  |  | Tire road wear particles sediment incubator test | | 0.2-1.3 ug/g | | LC/MS/MS | NA | |  |  | (Unice et al., 2015) | |
| 4-tert-butyl phenol | 98-54-4  (DTXSID1020221) | | antioxidant | | Cured tire tread material | | 2.1 ug/g | | LC/MS/MS | NA | | **H**/M | | (Unice et al., 2015) | |
| N-cyclohexylbenzothiazole-2-sulfenamide | 95-33-0  (DTXSID5020360) | | Accelerator | | Uncured tire tread material | | 1400 ug/g | | LC/MS/MS | NA | | **VH/VH** | | (Unice et al., 2015) | |
|  |  |  |  |  | Tire road wear particles leachate | | <0.01-<1 ug/g | | LC/MS/MS | NA | |  |  | (Unice et al., 2015) | |
|  |  |  |  |  | Tire road wear particles sediment incubator test | | <0.4-<3 ug/g | | LC/MS/MS | NA | |  |  | (Unice et al., 2015) | |
| n-Hexadecane | 544-76-3  (DTXSID0027195) | |  | | Tire crumb rubber | | 0.94-3.6 mg/kg | | GC/MS/MS | NA | | L/ND | | (US EPA, 2019) | |
| PAHs | | | | | | | | | | | | | | | |
| Acenaphthylene | 208-96-8  (DTXSID3023845) |  | | Field crumb rubber | | 0.02–0.49 μg/g | | GC-MS/MS | | | Portugal | | VH/VH | | (Celeiro et al., 2021) |
|  |  |  |  | Surface Runoff | | 3.04 ng/L | | GC-MS/MS | | | South Korea | |  |  | (Nguyen et al., 2021) |
| Anthracene | 120-12-7  (DTXSID0023878) |  | | Tire wear particles | | 2.27 μg/g | | GC-MS | | |  | | **VH/VH** | | (Halle et al., 2021) |
|  |  |  |  | Pristine tire particles | | 6.91 μg/g | | GC-MS | | |  | |  |  | (Halle et al., 2021) |
|  |  |  |  | Field crumb rubber | | 0.01–0.62 μg/g | | GC-MS/MS | | | Portugal | |  |  | (Celeiro et al., 2021) |
|  |  |  |  | Roadway particles | | 7.36 ppm | | GC/MS/MS | | |  | |  |  | (Kreider et al., 2010) |
| Benzo(a)anthracene | 56-55-3  (DTXSID5023902) |  | | Roadway particles | | 38.65 ppm | | GC/MS/MS | | |  | | **VH/VH** | | (Kreider et al., 2010) |
| Benzo[a]pyrene (BaP) | 50-32-8  (DTXSID2020139) |  | | Surface Runoff | | 1.04 ng/L | | GC-MS/MS | | | South Korea | | **VH/VH** | | (Nguyen et al., 2021) |
|  |  |  |  | Roadway particles | | 12.51 ppm | | GC/MS/MS | | |  | |  |  | (Kreider et al., 2010) |
|  |  |  |  | Tire particles | | 0.269-10.1 μg/g | | GC/MS/MS | | | Sweden | |  |  | (Sadiktsis et al., 2012) |
|  |  |  |  | Tire crumb rubber | | 0.74-0.78 mg/kg | | GC/MS/MS | | | NA | |  | | (US EPA, 2019) |
| Benzo[b]fluoranthene | 205-99-2  (DTXSID0023907) |  | | Tire particles | | 0.166-3.18 μg/g | | GC/MS/MS | | | Sweden | | **VH/VH** | | (Sadiktsis et al., 2012) |
| Benzo[g,h,i]perylene | 191-24-2  (DTXSID5023908) |  | | Tire particles | | 1.24-33.1 μg/g | | GC/MS/MS | | | Sweden | | *L/H* | | (Sadiktsis et al., 2012) |
|  |  |  |  | Tire crumb rubber | | 1.3 mg/kg | | GC/MS/MS | | | NA | |  |  | (US EPA, 2019) |
| benzo[k]fluoranthene | 207-08-9  (DTXSID0023909) |  | | Tire particles | | 0.0216-1.02 μg/g | | GC/MS/MS | | | Sweden | | **VH/VH** | | (Sadiktsis et al., 2012) |
| Coronene | 191-07-1  (DTXSID5047740) |  | | Tire particles | | 0.936-26.4 μg/g | | GC/MS/MS | | | Sweden | | *L/VH* | | (Sadiktsis et al., 2012) |
| Benzo[e]pyrene | 192-97-2  (DTXSID3023764) |  | | Tire particles | | 0.611-7.29 μg/g | | GC/MS/MS | | | Sweden | | **VH/VH** | | (Sadiktsis et al., 2012) |
| Dibenz[a,h]anthracene | 53-70-3  (DTXSID9020409) |  | | Tire particles | | 0.00856-0.146 μg/g | | GC/MS/MS | | | Sweden | | **VH/VH** | | (Sadiktsis et al., 2012) |
| Dibenzo[a,e]pyrene | 192-65-4  (DTXSID3052690) |  | | Tire particles | | 0.0120-0.392 μg/g | | GC/MS/MS | | | Sweden | | *L/VH* | | (Sadiktsis et al., 2012) |
| Dibenzo[a,h]pyrene | 189-64-0  (DTXSID4059752) |  | | Tire particles | | 0.00380-0.0675 μg/g | | GC/MS/MS | | | Sweden | | *L/VH* | | (Sadiktsis et al., 2012) |
| dibenzo[a,l]pyrene | 191-30-0  (DTXSID9059753) |  | | Tire particles | | 0.000929-0.0192 μg/g | | GC/MS/MS | | | Sweden | | *L/VH* | | (Sadiktsis et al., 2012) |
| dibenzo[a,i]pyrene | 189-55-9  (DTXSID9059751) |  | | Tire particles | | 0.0164-0.720 μg/g | | GC/MS/MS | | | Sweden | | *L/VH* | | (Sadiktsis et al., 2012) |
| Fluoranthene | 206-44-0  (DTXSID3024104) |  | | Field crumb rubber | | 0.3–9.4 μg/g | | GC-MS/MS | | | Portugal | | **VH/VH** | | (Celeiro et al., 2021) |
|  |  |  |  | Surface Runoff | | 10.4 ng/L | | GC-MS/MS | | | South Korea | |  |  | (Nguyen et al., 2021) |
|  |  |  |  | Roadway particles | | 82.13 ppm | | GC/MS/MS | | |  | |  |  | (Kreider et al., 2010) |
|  |  |  |  | Field Crumb Rubber | | 8 mg/kg | | GC/MS/MS | | | Norway | |  |  | (Halsband et al., 2020) |
|  |  |  |  | Commercial Crumb Rubber | | 7 mg/kg | | GC/MS/MS | | | NA | |  |  | (Halsband et al., 2020) |
|  |  |  | | Tire crumb rubber | | 4.5-6.1 mg/kg | | GC/MS/MS | | | NA | |  |  | (US EPA, 2019) |
| Indeno[1,2,3-cd]pyrene | 193-39-5  (DTXSID8024153) |  | | Tire particles | | 0.373-14.0 μg/g | | GC/MS/MS | | | Sweden | | VH/VH | | (Sadiktsis et al., 2012) |
| Indeno[1,2,3-cd]fluoranthene | 193-43-1  (DTXSID60172898) |  | | Tire particles | | 0.00121-0.102 μg/g | | GC/MS/MS | | | Sweden | | ND/ND | | (Sadiktsis et al., 2012) |
| Naphthalene | 91-20-3  (DTXSID8020913) |  | | Field crumb rubber | | 0.05–0.36 μg/g | | GC-MS/MS | | | Portugal | | **VH/VH** | | (Celeiro et al., 2021) |
|  |  |  | | Surface Runoff | | 21.4 ng/L | | GC-MS/MS | | | South Korea | |  |  | (Nguyen et al., 2021) |
| Phenanthrene | 85-01-8  (DTXSID6024254) |  | | Tire wear particles | | 5.32 μg/g | | GC-MS | | | NA | | **VH/VH** | | (Halle et al., 2021) |
|  |  |  |  | Pristine tire particles | | 17.06 μg/g | | GC-MS | | | South Korea | |  |  | (Halle et al., 2021) |
|  |  |  |  | Surface Runoff | | 40.6 ng/L | | GC-MS/MS | | | South Korea | |  |  | (Nguyen et al., 2021) |
|  |  |  |  | Field crumb rubber | | 0.07–4.30 μg/g | | GC-MS/MS | | | Portugal | |  |  | (Celeiro et al., 2021) |
|  |  |  |  | Roadway particles | | 54.3 ppm | | GC/MS/MS | | |  | |  |  | (Kreider et al., 2010) |
|  |  |  |  | Field Crumb Rubber | | 3.8-6.5 mg/kg | | GC/MS/MS | | | Norway | |  |  | (Halsband et al., 2020) |
|  |  |  |  | Commercial Crumb Rubber | | 5.9 mg/kg | | GC/MS/MS | | | NA | |  |  | (Halsband et al., 2020) |
|  |  |  |  | Tire crumb rubber | | 3.6-2.3 mg/kg | | GC/MS/MS | | | NA | |  |  | (US EPA, 2019) |
| Perylene | 198-55-0  (DTXSID4047753) |  | | Tire particles | | 0.104-1.11 μg/g | | GC/MS/MS | | | Sweden | | *L/VH* | | (Sadiktsis et al., 2012) |
| Picene | 213-46-7  (DTXSID8073895) |  | | Tire particles | | 0.0229-0.538 μg/g | | GC/MS/MS | | | Sweden | | *L/VH* | | (Sadiktsis et al., 2012) |
| Pyrene | 129-00-0  (DTXSID3024289) |  | | Tire wear particles | | 37.99 μg/g | | GC-MS | | | NA | | **VH/**L | | (Halle et al., 2021) |
|  |  |  |  | Pristine tire particles | | 29.99 μg/g | | GC-MS | | |  |  |  |  | (Halle et al., 2021) |
|  |  |  |  | Field crumb rubber | | 1.0–30 μg/g | | GC-MS/MS | | | Portugal | |  |  | (Celeiro et al., 2021) |
|  |  |  |  | Surface Runoff | | 8.77 ng/L | | GC-MS/MS | | | South Korea | |  |  | (Nguyen et al., 2021) |
|  |  |  |  | Roadway particles | | 54.84 ppm | | GC/MS/MS | | |  | |  |  | (Kreider et al., 2010) |
|  |  |  |  | Field Crumb Rubber | | 24-25 mg/kg | | GC/MS/MS | | | Norway | |  |  | (Halsband et al., 2020) |
|  |  |  |  | Commercial Crumb Rubber | | 24 mg/kg | | GC/MS/MS | | | NA | |  |  | (Halsband et al., 2020) |
|  |  |  |  | Tire crumb rubber | | 12-18 mg/kg | | GC/MS/MS | | | NA | |  |  | (US EPA, 2019) |
| Metals | | | | | | | | | | | | | | | |
| Aluminum | 7429-90-5  (DTXSID3040273) |  | | Tire wear particles | | 324.97 μg/g | | ICP-MS | | |  | | **VH/**VH | | (Halle et al., 2021) |
|  |  |  |  | Pristine tire particles | | 521.75 μg/g | |  |  |  |  |  |  |  |  |
|  |  |  |  | Freshwater tire particle leachate | | 439 μg/L | | ICP-MS | | |  | |  |  | (Capolupo et al., 2020) |
|  |  |  |  | Marine tire particle leachate | | 884 μg/L | |  |  |  |  |  |  |  |  |
| Arsenic | 7440-38-2  (DTXSID4023886) |  | | Tire crumb rubber | | 0.20-0.20 mg/kg | | ICP-MS | | | NA | | **VH/VH** | | (US EPA, 2019) |
| Antimony | 7440-36-0  (DTXSID5023879) |  | | Field Crumb Rubber | | 0.24-4.39 mg/kg | | ICP-MS | | | Norway | | **M**/VH | | (Halsband et al., 2020) |
|  |  |  |  | Field Crumb Rubber Seawater Leachate | | <0.2-1 µg/L | |  |  |  |  |  |  |  |  |
|  |  |  |  | Commercial Crumb Rubber | | 4.88-6.7 mg/kg | | ICP-MS | | | NA | |  |  | (Halsband et al., 2020) |
|  |  |  | | Commercial Crumb Rubber Seawater Leachate | | 0.25-1.8 µg/L | |  |  |  |  |  |  |  |  |
| Cadmium | 7440-43-9  (DTXSID1023940) |  | | Tire wear particles | | 0.82 μg/g | | ICP-MS | | | NA | | **VH**/**VH** | | (Halle et al., 2021) |
|  |  |  |  | Pristine tire particles | | 0.91 μg/g | |  |  |  |  |  |  |  |  |
|  |  |  |  | Field Crumb Rubber | | 0.83-1.84 mg/kg | | ICP-MS | | | Norway | |  |  | (Halsband et al., 2020) |
|  |  |  |  | Field Crumb Rubber Seawater Leachate | | <0.2-1.5 µg/L | |  |  |  |  |  |  |  |  |
|  |  |  |  | Commercial Crumb Rubber | | 0.93-1.36 mg/kg | | ICP-MS | | | NA | |  |  | (Halsband et al., 2020) |
|  |  |  |  | Commercial Crumb Rubber Seawater Leachate | | <0.2-2.2 µg/L | |  |  |  |  |  |  |  |  |
|  |  |  |  | Tire crumb rubber | | 0.55-0.95 mg/kg | | ICP-MS | | | NA | |  |  | (US EPA, 2019) |
| Cobalt | 7440-48-4  (DTXSID1031040) |  | | Freshwater tire particle leachate | | 11.9 μg/L | | ICP-MS | | |  | | **H**/**L** | | (Capolupo et al., 2020) |
|  |  |  |  | Marine tire particle leachate | | 13.0 μg/L | | ICP-MS | | |  |  |  |  | (Capolupo et al., 2020) |
|  |  |  |  | Field Crumb Rubber | | 36.5-200 mg/kg | | ICP-MS | | | Norway | |  |  | (Halsband et al., 2020) |
|  |  |  |  | Field Crumb Rubber Seawater Leachate | | <2-57 μg/L | |  |  |  |  |  |  |  |  |
|  |  |  |  | Commercial Crumb Rubber | | 66-84 mg/kg | | ICP-MS | | | NA | |  |  | (Halsband et al., 2020) |
|  |  |  |  | Commercial Crumb Rubber Seawater Leachate | | <2-13.4 μg/L | |  |  |  |  |  |  |  |  |
|  |  |  |  | Tire crumb rubber | | 140-190 mg/kg | | ICP-MS | | | NA | |  |  | (US EPA, 2019) |
| Chromium | 7440-47-3  (DTXSID3031022) |  | | Tire wear particles | | 23.72 μg/g | | ICP-MS | | | NA | | **VH**/**M** | | (Halle et al., 2021) |
|  |  |  |  | Pristine tire particles | | 36.45 μg/g | | ICP-MS | | |  |  |  |  | (Halle et al., 2021) |
|  |  |  |  | Freshwater tire particle leachate | | 1.53 μg/L | | ICP-MS | | | NA | |  |  | (Capolupo et al., 2020) |
|  |  |  |  | Marine tire particle leachate | | 4.5 μg/L | | ICP-MS | | |  |  |  |  | Capolupo et al., 2020) |
|  |  |  |  | Field Crumb Rubber | | 1.9-5.5 mg/kg | | ICP-MS | | | Norway | |  |  | (Halsband et al., 2020) |
|  |  |  |  | Field Crumb Rubber Seawater Leachate | | 4-10.6 μg/L | |  |  |  |  |  |  |  |  |
|  |  |  |  | Commercial Crumb Rubber | | 2.11-2.93 mg/kg | | ICP-MS | | | NA | |  |  | (Halsband et al., 2020) |
|  |  |  |  | Commercial Crumb Rubber Seawater Leachate | | 3.7-5 μg/L | |  |  |  |  |  |  |  |  |
|  |  |  |  | Tire crumb rubber | | 1.6-1.8 mg/kg | | ICP-MS | | | NA | |  |  | (US EPA, 2019) |
| Copper | 7440-50-8  (DTXSID2023985) |  | | Tire wear particles | | 54.82 μg/g | | ICP-MS | | | NA | | **VH**/**VH** | | (Halle et al., 2021) |
|  |  |  |  | Pristine tire particles | | 25.51 μg/g | | ICP-MS | | |  |  |  |  |  |
|  |  |  |  | Freshwater tire particle leachate | | 6.4 μg/L | | ICP-MS | | | NA | |  |  | (Capolupo et al., 2020) |
|  |  |  |  | Marine tire particle leachate | | 44 μg/L | | ICP-MS | | |  |  |  |  |  |
|  |  |  |  | Field Crumb Rubber | | 17.7-85.1 mg/kg | | ICP-MS | | | Norway | |  |  | (Halsband et al., 2020) |
|  |  |  |  | Field Crumb Rubber Seawater Leachate | | 28-66 μg/L | |  |  |  |  |  |  |  |  |
|  |  |  |  | Commercial Crumb Rubber | | 6.7-22.6 mg/kg | | ICP-MS | | | NA | |  |  | (Halsband et al., 2020) |
|  |  |  |  | Commercial Crumb Rubber Seawater Leachate | | 18-49 μg/L | |  |  |  |  |  |  |  |  |
| Iron | 7439-89-6  (DTXSID5043710) |  | | Tire wear particles | | 805.52 μg/g | | ICP-MS | | | NA | | **L**/**L** | | (Halle et al., 2021) |
|  |  |  |  | Pristine tire particles | | 953.61 μg/g | |  |  |  |  |  |  |  |  |
|  |  |  |  | Field Crumb Rubber | | 729-1214 mg/kg | | ICP-MS | | | Norway | |  |  | (Halsband et al., 2020) |
|  |  |  |  | Field Crumb Rubber Seawater Leachate | | 20-377 μg/L | |  |  |  |  |  |  |  |  |
|  |  |  |  | Commercial Crumb Rubber | | 349-562 mg/kg | | ICP-MS | | | NA | |  |  | (Halsband et al., 2020) |
|  |  |  |  | Commercial Crumb Rubber Seawater Leachate | | 23-126 μg/L | |  |  |  |  |  |  |  |  |
| Lead | 7439-92-1  (DTXSID2024161) |  | | Tire wear particles | | 7.39 μg/g | | ICP-MS | | | NA | | **VH**/**VH** | | (Halle et al., 2021) |
|  |  |  |  | Pristine tire particles | | 7.92 μg/g | |  |  |  |  |  |  |  |  |
|  |  |  |  | Freshwater tire particle leachate | | 0.59 μg/L | | ICP-MS | | | NA | |  |  | (Capolupo et al., 2020) |
|  |  |  |  | Marine tire particle leachate | | 2.28 μg/L | |  |  |  |  |  |  |  |  |
|  |  |  |  | Field Crumb Rubber | | 17.8-24.9 mg/kg | | ICP-MS | | | Norway | |  |  | (Halsband et al., 2020) |
|  |  |  |  | Field Crumb Rubber Seawater Leachate | | 1.7-7.3 μg/L | |  |  |  |  |  |  |  |  |
|  |  |  |  | Commercial Crumb Rubber | | 16.6-28.6 mg/kg | | ICP-MS | | | NA | |  |  | (Halsband et al., 2020) |
|  |  |  |  | Commercial Crumb Rubber Seawater Leachate | | 1.6-3.8 μg/L | |  |  |  |  |  |  |  |  |
|  |  |  |  | Tire crumb rubber | | 13-24 mg/kg | | ICP-MS | | | NA | |  |  | (US EPA, 2019) |
| Manganese | 7439-96-5  (DTXSID2024169) |  | | Field Crumb Rubber | | 15.6 mg/kg | | ICP-MS | | | Norway | | **M**/M | | (Halsband et al., 2020) |
|  |  |  |  | Field Crumb Rubber Seawater Leachate | | <2-79 μg/L | |  |  |  |  |  |  |  |  |
|  |  |  |  | Commercial Crumb Rubber | | 4.4-5.12 mg/kg | | ICP-MS | | | NA | |  |  | (Halsband et al., 2020) |
|  |  |  |  | Commercial Crumb Rubber Seawater Leachate | | <2-54 μg/L | |  |  |  |  |  |  |  |  |
| Nickel | 7440-02-0  (DTXSID2020925) |  | | Field Crumb Rubber | | 2.56-4.3 mg/kg | | ICP-MS | | | Norway | | **VH**/**H** | | (Halsband et al., 2020) |
|  |  |  |  | Field Crumb Rubber Seawater Leachate | | <2-4 μg/L | |  |  |  |  |  |  |  |  |
|  |  |  |  | Commercial Crumb Rubber | | 2.8-3.6 mg/kg | | ICP-MS | | | NA | |  |  | (Halsband et al., 2020) |
|  |  |  |  | Commercial Crumb Rubber Seawater Leachate | | <2-4 μg/L | |  |  |  |  |  |  |  |  |
| Zinc | 7440-66-6  (DTXSID7035012) |  | | Tire wear particles | | 8298.70 μg/g | | ICP-MS | | | NA | | **VH**/**VH** | | (Halle et al., 2021) |
|  |  |  |  | Pristine tire particles | | 8644.05 μg/g | | ICP-MS | | |  |  |  |  | (Halle et al., 2021) |
|  |  |  |  | Freshwater tire particle leachate | | 12,130 μg/L | | ICP-MS | | |  |  |  |  | (Capolupo et al., 2020) |
|  |  |  |  | Marine tire particle leachate | | 5,138 μg/L | |  |  |  |  |  |  |  |  |
|  |  |  |  | Field Crumb Rubber | | 12544-22601 mg/kg | | ICP-MS | | | Norway | |  |  | (Halsband et al., 2020) |
|  |  |  |  | Field Crumb Rubber Seawater Leachate | | 0.21-22.4 μg/L | |  |  |  |  |  |  |  |  |
|  |  |  |  | Commercial Crumb Rubber | | 14136-15399 mg/kg | | ICP-MS | | | NA | |  |  | (Halsband et al., 2020) |
|  |  |  |  | Commercial Crumb Rubber Seawater Leachate | | 0.13-4.11 μg/L | |  |  |  |  |  |  |  |  |
|  |  |  |  | Tire crumb rubber | | 15000-17000 mg/kg | | ICP-MS | | | NA | |  |  | (US EPA, 2019) |
| Plasticizers | | | | | | | | | | | | | | | |
| Acetophenone | 98-86-2  (DTXSID6021828) |  | | Freshwater tire particle leachate | | 22 μg/L | | GC-MS | | | NA | | **L**/ND | | (Capolupo et al., 2020) |
|  |  |  |  | Marine tire particle leachate | | 13.2 μg/L | | GC-MS | | |  |  |  |  |  |
| *n*-cyclohexylformamide | 766-93-8  (DTXSID40227416) |  | | Freshwater tire particle leachate | | 1,059 μg/L | | GC-MS | | | NA | | *M*/ND | | (Capolupo et al., 2020) |
|  |  |  |  | Marine tire particle leachate | | 788 μg/L | | GC-MS | | |  |  |  |  |  |
| Phthalide | 87-41-2  (DTXSID0052594) |  | | Freshwater tire particle leachate | | 5.5 μg/L | | GC-MS | | | NA | | L/ND | | (Capolupo et al., 2020) |
|  |  |  |  | Marine tire particle leachate | | 2.4 μg/L | | GC-MS | | |  |  |  |  |  |
|  |  |  |  | Field Crumb Rubber | | 0.4-78 mg/kg | | GC/MS/MS | | | Norway | |  |  | (Halsband et al., 2020) |
|  |  |  |  | Commercial Crumb Rubber | | 0.10 mg/kg | | GC/MS/MS | | | NA | |  |  | (Halsband et al., 2020) |
| Bisphenol A (BPA) | 80-05-7  (DTXSID7020182) |  | | Freshwater tire particle leachate | | 8.6 μg/L | | GC-MS | | | NA | | **H**/**VH** | | (Capolupo et al., 2020) |
|  |  |  |  | Marine tire particle leachate | | 6.4 μg/L | | GC-MS | | |  |  |  |  | Capolupo et al., 2020) |
|  |  |  |  | Field Crumb Rubber | | 1.1-4 mg/kg | | GC/MS/MS | | | Norway | |  |  | (Halsband et al., 2020) |
|  |  |  |  | Commercial Crumb Rubber | | 3 mg/kg | | GC/MS/MS | | | NA | |  |  | (Halsband et al., 2020) |
| Dimethyl Adipate | 627-93-0  (DTXSID8025096) |  | | Field crumb rubber | | 0.01–0.02 μg/g | | GC-MS/MS | | | Portugal | | M/ND | | (Celeiro et al., 2021) |
| Diethyl phthalate | 84-66-2  (DTXSID7021780) |  | | Field crumb rubber | | 0.03–0.70 μg/g | | GC-MS/MS | | | Portugal | | **M**/**M** | | (Celeiro et al., 2021) |
| Diisobutyl phthalate | 84-69-5  (DTXSID9022522) |  | | Field crumb rubber | | 0.2–29 μg/g | | GC-MS/MS | | | Portugal | | **VH/**VH | | (Celeiro et al., 2021) |
| Dibutyl phthalate | 84-74-2  (DTXSID2021781) |  | | Field crumb rubber | | 0.1–11 μg/g | | GC-MS/MS | | | Portugal | | **VH/H** | | (Celeiro et al., 2021) |
|  |  |  |  | Tire crumb rubber | | 0.68-1.5 mg/kg | | GC/MS/MS | | | NA | |  |  | (US EPA, 2019) |
| Benzyl butyl phthalate | 85-68-7  (DTXSID3020205) |  | | Field crumb rubber | | 0.04–1.4 μg/g | | GC-MS/MS | | | Portugal | | **VH**/**VH** | | (Celeiro et al., 2021) |
| Dimethyl phthalate | 131-11-3  (DTXSID3022455) |  | | Field crumb rubber | | 0.01–0.06 μg/g | | GC-MS/MS | | | Portugal | | **M**/**M** | | (Celeiro et al., 2021) |
| Di(2-ethylhexyl)phthalate | 117-81-7  (DTXSID5020607) |  | | Field crumb rubber | | 6.2–59 μg/g | | GC-MS/MS | | | Portugal | | **H**/**VH** | | (Celeiro et al., 2021) |
| Di-n-octyl phthalate | 117-84-0  (DTXSID1021956) |  | | Field crumb rubber | | 0.51μg/g | | GC-MS/MS | | | Portugal | | H/ND | | (Celeiro et al., 2021) |

**SUPPLEMENTARY INFORMATION REFERENCES**

Cao, G., Wang, W., Zhang, J., Wu, P., Zhao, X., Yang, Z., Hu, D., Cai, Z., 2022. New Evidence of Rubber-Derived Quinones in Water, Air, and Soil. Environ. Sci. Technol. 56, 4142–4150. https://doi.org/10.1021/acs.est.1c07376

Capolupo, M., Sorensen, L., Jayasena, K.D.R., Booth, A.M., Fabbri, E., 2020. Chemical composition and ecotoxicity of plastic and car tire rubber leachates to aquatic organisms. Water Res 169, 115270. https://doi.org/10.1016/j.watres.2019.115270

Challis, J.K., Popick, H., Prajapati, S., Harder, P., Giesy, J.P., McPhedran, K., Brinkmann, M., 2021. Occurrences of Tire Rubber-Derived Contaminants in Cold-Climate Urban Runoff. Environ. Sci. Technol. Lett. 8, 961–967. https://doi.org/10.1021/acs.estlett.1c00682

Halle, L.L., Palmqvist, A., Kampmann, K., Jensen, A., Hansen, T., Khan, F.R., 2021. Tire wear particle and leachate exposures from a pristine and road-worn tire to Hyalella azteca: Comparison of chemical content and biological effects. Aquatic Toxicology 232, 105769. https://doi.org/10.1016/j.aquatox.2021.105769

Halsband, C., Sørensen, L., Booth, A.M., Herzke, D., 2020. Car Tire Crumb Rubber: Does Leaching Produce a Toxic Chemical Cocktail in Coastal Marine Systems? Frontiers in Environmental Science 8.

Johannessen, C., Helm, P., Metcalfe, C.D., 2021. Detection of selected tire wear compounds in urban receiving waters. Environmental Pollution 287, 117659. https://doi.org/10.1016/j.envpol.2021.117659

Kreider, M.L., Panko, J.M., McAtee, B.L., Sweet, L.I., Finley, B.L., 2010. Physical and chemical characterization of tire-related particles: comparison of particles generated using different methodologies. Sci Total Environ 408, 652–659. https://doi.org/10.1016/j.scitotenv.2009.10.016

Peter, K.T., Tian, Z., Wu, C., Lin, P., White, S., Du, B., McIntyre, J.K., Scholz, N.L., Kolodziej, E.P., 2018. Using High-Resolution Mass Spectrometry to Identify Organic Contaminants Linked to Urban Stormwater Mortality Syndrome in Coho Salmon. Environ. Sci. Technol. 52, 10317–10327. https://doi.org/10.1021/acs.est.8b03287

Rauert, C., Charlton, N., Okoffo, E.D., Stanton, R.S., Agua, A.R., Pirrung, M.C., Thomas, K.V., 2022. Concentrations of Tire Additive Chemicals and Tire Road Wear Particles in an Australian Urban Tributary. Environ. Sci. Technol. 56, 2421–2431. https://doi.org/10.1021/acs.est.1c07451

Sadiktsis, I., Bergvall, C., Johansson, C., Westerholm, R., 2012. Automobile Tires—A Potential Source of Highly Carcinogenic Dibenzopyrenes to the Environment. Environ. Sci. Technol. 46, 3326–3334. https://doi.org/10.1021/es204257d

Unice, K.M., Bare, J.L., Kreider, M.L., Panko, J.M., 2015. Experimental methodology for assessing the environmental fate of organic chemicals in polymer matrices using column leaching studies and OECD 308 water/sediment systems: Application to tire and road wear particles. Science of The Total Environment 533, 476–487. https://doi.org/10.1016/j.scitotenv.2015.06.053
